# Genomic and biochemical comparison of allelic triple‐mutant lines derived from conventional breeding and multiplex gene editing

**DOI:** 10.1002/tpg2.70056

**Published:** 2025-06-05

**Authors:** Junqi Liu, Ritesh Kumar, Samatha Gunapati, Steven Mulkey, Yinjie Qiu, Yer Xiong, Vishnu Ramasubramanian, Jean‐Michel Michno, Praveen Awasthi, Daniel D. Gallaher, Thi Thao Nguyen, Won‐Seok Kim, Hari B. Krishnan, Aaron J. Lorenz, Robert M. Stupar

**Affiliations:** ^1^ Department of Agronomy and Plant Genetics University of Minnesota Saint Paul Minnesota USA; ^2^ Ball Horticultural Company West Chicago Illinois USA; ^3^ Department of Biology Washington University St. Louis Missouri USA; ^4^ Minnesota Supercomputing Institute University of Minnesota Minneapolis Minnesota USA; ^5^ Department of Food Science and Nutrition University of Minnesota Saint Paul Minnesota USA; ^6^ Gehrke Proteomics Center, Christopher S. Bond Life Sciences Center University of Missouri Columbia Missouri USA; ^7^ Division of Plant Science and Technology University of Missouri Columbia Missouri USA; ^8^ US Department of Agriculture‐Agricultural Research Service Plant Genetics Research Unit Columbia Missouri USA

## Abstract

Multiplex gene editing allows for the simultaneous targeting and mutagenesis of multiple loci in a genome. This tool is particularly valuable for plant genetic improvement, as plant genomes often require mutations at multiple loci to confer useful and/or novel traits. However, the regulation of gene editing can vary depending on the number of loci targeted. In this study, we developed triple‐mutant soybean (*Glycine max* (L.) Merrill) lines using different crop improvement strategies, including conventional backcross breeding of standing variant alleles and clustered regularly interspaced short palindromic repeats‐based multiplex editing to introduce new alleles. The mutations were targeted to genes encoding seed antinutritional components, as previously described in a *triple null* soybean carrying knockout alleles for a Kunitz trypsin inhibitor, a soybean agglutinin, and the allergen P34 protein. The products developed from these respective genetic improvement pipelines were tested for differences between the triple‐mutant lines and their parental lines. Analyses included genomics, seed proteomics, trypsin inhibition, seed protein digestibility, and harvestable yield of the different lines. We observed that both multiplex gene editing and conventional breeding approaches produced essentially equivalent products in comparison to their parental lines. We conclude that the multiplex gene editing strategy is not inherently riskier than conventional breeding for developing complex mutant lines of this type.

## Core Ideas

- Plant lines with novel traits/mutations can be developed using different genetic approaches.
- Historically, host‐repaired gene edits have been differentially regulated based on the number of targeted loci.
- Targeting multiple loci via gene editing is analogous to stacking mutations via conventional backcross breeding.
- The plant products developed using these two approaches are functionally equivalent.

**Abbreviations**
Chr: chromosome
CRISPR: clustered regularly interspaced short palindromic repeats
DDA: data‐dependent acquisition
DEP: differentially expressed proteins
DIA: data‐independent acquisition
DSB: double‐stranded break
DTT: dithiothreitol
FA: formic acid
hph: high‐pH
IAA: iodoacetamide
IM: ion mobility
KTi: Kunitz trypsin inhibitor
LE: lectin
PASEF: parallel accumulation–serial fragmentation
SDN: site‐directed nuclease
SNP: single‐nucleotide polymorphism
UMGC: University of Minnesota Genomic Center
WGS: whole genome sequencing
Wm82: Williams 82
WPT: whole plant transformation

## 1 INTRODUCTION

Gene editing approaches like clustered regularly interspaced short palindromic repeats (CRISPR)/Cas offer a toolbox for crop improvement. These tools include a range of site‐directed nuclease (SDN) applications, the simplest being defined as SDN‐1, wherein induced double‐stranded breaks (DSB) are repaired by the cell's endogenous repair pathways to produce new mutations (e.g., single‐nucleotide polymorphisms (SNPs) or small insertions or deletions). SDN‐1 mutagenesis tools have quickly been adopted by the crop science research community to generate a suite of proof‐of‐concept crop lines with enhanced disease resistances (Gao et al., 2024; Oliva et al., 2019), abiotic stress tolerances (Shaheen et al., 2023), and quality traits (Demorest et al., 2016; Satterlee et al., 2024; J. Zhang et al., 2024). Realizing the potential utility of gene editing assumes that the tools are accessible to researchers and products are affordable to bring to the marketplace. The affordability of bringing such products to market is intrinsically linked to the regulatory status of such products, which is continuously evolving and highly disparate across geographic regions and political structures.

The regulatory guidelines in the United States of America provide an interesting example, wherein SDN‐1 events have been differentially regulated at times in recent history depending on the number of DSBs that occur in the product development pipeline. Plant materials with mutation(s) derived from DSBs below the threshold number have been unregulated and treated the same as natural variants, provided the mutation does not confer unique pesticide traits or morphological phenotypes with altered form or function. However, materials with mutations derived from DSBs above the threshold number have been subject to regulation by the United States Department of Agriculture division of Animal and Plant Health Inspection Agency (USDA‐APHIS). This paradigm of regulation implicitly assumes that plants with more targeted mutations are riskier products and thus require greater scrutiny than those with fewer such mutations. The majority of crop species have high frequencies of gene duplication, particularly polyploid species (e.g., wheat, sugarcane, strawberry) and paleopolyploid species like soybean (*Glycine max* (L.) Merrill) (Schmutz et al., 2010). Thus, the nature of crop genomes dictates that many traits cannot be altered for beneficial variants unless multiple loci (e.g., homoeologous gene duplications) are targeted in combination. This point is well illustrated by early gene editing studies focusing on soybean fatty acid composition (Haun et al., 2014) and wheat disease resistance (Y. Zhang et al., 2017). Furthermore, one of the most important advantages of CRISPR‐based gene editing is that it also allows for the simultaneous targeting of non‐homologous and non‐homoeologous genes, a strategy commonly known as multiplex gene editing. This provides researchers the ability to target combinations of gene knockouts that may enhance trait variation beyond the scope of what can be accomplished by targeting only a single gene.

It is worth questioning the notion of whether multiplex gene editing should be differentially regulated compared to single gene editing. Is there any basis to consider a product generated by multiplex gene editing as fundamentally different from an analogous product developed through single gene editing, conventional breeding, or any other method? Is there any reason to think such a product would be less safe or any less useful?

To address these questions, we have developed analogous triple‐mutant soybean lines using two different strategies: (1) conventional backcross introgression of standing variants and (2) de novo generation of novel mutant alleles through CRISPR/Cas9 gene editing. The target genes for this study are based on previous work stacking three standard variants into a common background of soybean line Williams 82 (Schmidt et al., 2015). The three alleles encode mutant forms of a lectin (LE) gene, a Kunitz trypsin inhibitor (KTi3), and the most abundant protease‐like allergen P34. The functional forms of these alleles encode major anti‐nutrients for soybean seeds when utilized for animal feed or human consumption (Liener, 1994).

In this study, parallel projects were performed to develop new triple‐mutant lines for the LE, KTi3, and P34 genes using a standard conventional breeding strategy and a multiplex gene editing strategy. Our analysis of the seed composition traits and the agronomic performance of the resulting lines offers an “apples‐to‐apples” comparison of these methodologies and provides insight into the whether unanticipated risks are conferred by the multiplex gene editing approach compared to the conventional breeding methodology.

## 2 MATERIALS AND METHODS

### 2.1 Development of triple‐mutant lines using conventional breeding

Natural variant alleles of the LE (gene model Glyma.02G012600), KTi3 (Glyma.08G341500), and P34 (Glyma.08G116300) genes (Schmidt et al., 2015) were, respectively, backcrossed into two different modern elite lines adapted to northern latitudes, M07‐292111 and MN0811CN. Four generations of backcrossing were used, followed by self‐pollination of the resulting BC_4_F_1_ plants to obtain BC_4_F_2_ seeds. Plants from BC_4_F_2_ seeds were genotyped using molecular markers to efficiently select plants that carried the mutations for the three genes in the M07‐292111 and MN0811CN backgrounds, respectively. Ten plants were selected for each background and were grown to produce BC_4_F_2:3_ seed. These BC_4_F_2:3_ seeds were planted in a “plant‐to‐row” manner in the summer of 2019. Seeds were bulked to create BC_4_F_2:4_ families. Two families in the M07‐292111 background were selected as triple‐mutant lines, designated as M07‐292111‐BC4TN1 and M07‐292111‐BC4TN2. One family in the MN0811CN background was selected as a triple‐mutant line, designated as M0811CN‐BC4TN1. These new families were considered near‐isogenic to their recurrent parent backgrounds and were entered into yield trials in 2020 and 2021.

**Core Ideas**

- Plant lines with novel traits/mutations can be developed using different genetic approaches.
- Historically, host‐repaired gene edits have been differentially regulated based on the number of targeted loci.
- Targeting multiple loci via gene editing is analogous to stacking mutations via conventional backcross breeding.
- The plant products developed using these two approaches are functionally equivalent.

### 2.2 Development of CRISPR triple‐mutant lines

For routine molecular cloning procedures, DH5α and TOP10 One‐Shot competent cells (Invitrogen Competent Cells, Thermo Fisher Scientific, Inc.) were transformed with various plasmids and selected on Luria‐Bertani (LB) agar plates supplemented with appropriate antibiotics. A disarmed *Agrobacterium rhizogenes* strain (18r12) was used for soybean whole plant transformation (WPT) as described in previously reported protocols (Liu et al., 2019; Paz et al., 2006, 2004). Both modular plasmid vectors (pMOD_A0521, pMOD_2103, and pMOD_C2906) and the destination T‐DNA vector (pTRANS_230d) were provided by the Daniel Voytas Lab (Čermák et al., 2017) and are also available via http://www.addgene.org.

The full‐length coding sequences of three target genes (LE: lectin Glyma.02G012600; KTi3: trypsin inhibitor Glyma.08G341500; P34 allergen: Glyma.08G116300) were retrieved from the Phytozome website: https://phytozome.jgi.doe.gov/pz/portal.html. The coding sequences were used to identify multiple CRISPR targets. Selection of targets was aided by online web tools designed to identify gRNAs with NGG as the PAM site and under the control of the *Arabidopsis thaliana* U6 promoter. The web tools we used are no longer available online, but CRISPR‐P 2.0 (http://crispr.hzau.edu.cn/cgi‐bin/CRISPR2/CRISPR) is a readily available platform for designing gRNAs with different PAM sites under the control of either U6 or U3 promoters.

Three assembled gRNA cassettes, each with three gRNAs (one per gene target), were subcloned into the destination T‐DNA vector pTRANS_230d (Čermák et al., 2017). The recombinant pTRANS_230d includes the following components: (1) a polycistronic expression cassette of three gRNAs under the control of the *Cestrum yellow leaf curling virus* (CmYLCV) promoter; (2) the Csy4:Cas9 fusion gene driven by a soybean ubiquitin promoter (promoter name abbreviated as “GmUbi”; Chiera et al., 2007); (3) a constitutively expressed exonuclease gene, TREX2, to facilitate the formation of longer deletions at the double strand breaks; (4) a BAR gene under the control of the CaMV35 promoter to confer resistance to glufosinate, a selective agent in the course of tissue culture for WPT. The version of Cas9 used in these experiments (abbreviated as AtCas9) was codon‐optimized for use in *Arabidopsis thaliana* (Fauser et al., 2014). The functional mechanism of this multiplexing vector system requires that individual single gRNAs are released from a primary RNA transcript upon cleavage by the Csy4 ribozyme.

The subcloning of the gRNA cassettes into pTRANS_230d was performed as follows. Three CRISPR targets per gene were identified, and all gRNA spacers were amplified from the modular plasmid pMOD_B2103 with corresponding primer set combinations (Tables S1 and S2). Briefly, each PCR reaction was composed of 1 µL pMOD_B2103 plasmid DNA (1 ng/µL), 2 µL of each primer (100 µM), 5 µL 10x KOD buffer, 5 µL 10 mM dNTP, 5 µL 5 mM MgSO4, 1 µL KOD DNA Polymerase, and 29 µL sterile water. Subsequently, an equal molar ratio of gRNA spacers was mixed with pMOD_B2103 plasmid as follows: 1.0 µL 10x diluted gRNA spacers (from each of four reactions); 10 µL 2x T7 ligase buffer; 1.0 µL Sap I; 1.0 µL Esp 3I; 1.0 µL T7 DNA ligase, for a thermal cycling of digestion/ligation reactions with 20 cycles × (5 min at 37°C, 10 min at 25°C). For final assembly of different modules into the destination T‐DNA vector, different amounts of four plasmids (pMOD_A0521, pMOD_B2103_3plex, pMOD_C2906, and pTRANS_MOD230d) were added to the following digestion/ligation reactions: 1 µL (50 ng/µL) pTRANS_230d, 1 µL (150 ng/µL) pMOD_A0521, 1 µL (150 ng/µL) pMOD_B2103_3plex, 1 µL (150 ng/µL) pMOD_C2906, 0.4 µL Aar I oligo, 0.5 µL Aar I, 1.0 µL T4 DNA ligase, 2.0 µL 10x T4 DNA ligase buffer, and 12 µL sterile water. Thermal cycling of digestion/ligation was set for 20 cycles × (5 min at 37°C, 10 min at 16°C). The resultant T‐DNA plasmids each carried three gRNA, one for each respective gene target. The T‐DNA plasmids were transformed into *A. rhizogenes* (18r12) strain in preparation for WPT.

Half‐seed explants were co‐cultivated with the disarmed *A. rhizogenes* strain (18r12) harboring T‐DNA vectors. Soybean WPT in the genotype “Bert” was carried out as previously described (Liu et al., 2019; Paz et al., 2006, 2004) using 5 mg/L glufosinate as a selection agent in the culture medium during the shoot induction and shoot elongation stages of growth. For optimal recovery of plantlets from rooting media, plants were initially transplanted to a growth chamber (16‐h light/8‐h dark photoperiodic conditions at 28°C) and maintained for approximately 14 days prior to final transplanting to larger pots (in 8 in. diameter) in a greenhouse.

### 2.3 Detection of transgene and mutations

After the tissue culture process of shoot induction, shoot elongation, selection, and root induction, putatively transgenic T_0_ plants were PCR‐screened for the presence of transgenes and mutations. First, genomic DNA was extracted using the QIAGEN Plant Mini KIT from the leaves of individual T_0_ plants. To identify transgene‐positive plants, different primer sets from the Gmubi/AtCAS9 junction, multiplex CRISPR targets, and/or the BAR gene were used for PCR amplification. The target regions were amplified by PCR with specific primers flanking each CRISPR target (Table S3), and an aliquot of the PCR products was separated by electrophoresis on (1x tris‐acetate‐EDTA) 1.5% agarose gels.

Detection of multiple mutations in somatic tissues of the T_0_ plants was performed using PCR heteroduplex assays (Zhu et al., 2014). Briefly, regions flanking the target regions were PCR‐amplified, denatured at 95°C for 5 min, and then renatured at room temperature for 10 min. 2 µL 6x Novex loading buffer was added to each sample prior to gel loading. Samples were run in precast 4%–20% gradient polyacrylamide gels in 1x tris‐borate‐EDTA (Thermo Fisher Scientific) at 200 V for 2 h for optimal resolution. Heteroduplex DNA with altered mobilities was visualized under UV light after a staining and destaining process.

For plants with putative mutations, PCR products encompassing the targeted region were purified and either sequenced directly or subcloned into pGEM‐T‐easy vector (Promega Corporation) and individual clones were sequenced separately. Sanger sequencing of PCR products and plasmids were carried out by the University of Minnesota Genomic Center (UMGC). Mutations were identified by performing multi‐alignments of the WT reference genome sequence and various mutant sequences at http://multalin.toulouse.inra.fr/multalin/.

### 2.4 Whole‐genome sequencing and data analysis

Whole‐genome sequencing of the T_0_ plant WPT673‐7 and T_2_ progeny plants WPT673‐7‐8‐8 and WPT673‐7‐12‐5, all carrying triple‐knockout mutations, was performed and compared to the untransformed parent line Bert. DNA from young leaves at the second trifoliate stage was extracted with the DNeasy plant mini kit (Qiagen). Whole genome resequencing was performed to approximately 20x genome coverage per sample at the UMGC using Illumina NovaSeq X platform. Adaptors sequences were removed using trimmomatic (v 0.39) (Bolger et al., 2014). Processed reads were mapped to the Wm82.a6 (where Wm82 is Williams 82) reference assembly (Espina et al., 2024) (Gmax_Wm82_a6_v1 genome assembly obtained from https://phytozome‐next.jgi.doe.gov/info/Gmax_Wm82_a6_v1) using bwa (0.7.17.CentOS7) aligner (Li & Durbin, 2009). Picard tool (v 2.25.6) (https://broadinstitute.github.io/picard/) was used to mark PCR duplicates prior to downstream variant calling. Identification of the transgene presence and chromosome location or absence was done as described (Virdi et al., 2020). Briefly, resequencing reads were aligned to the vector backbone and transgene sequence using the bash script TransGeneMap.sh (https://github.com/MeeshCompBio/Soybean_Scripts). Alignments were inspected via IGV browser. “Orphan reads,” read sequences in which the paired read maps to the vector sequence but the mate read does not, were mapped to the soybean reference genome (Wm82.a6.v1) using a script modified from Srivastava et al. (2014) (https://github.com/MeeshCompBio/Soybean_Scripts/blob/master/Orphan.pl).

Whole‐genome re‐sequencing of MN0811CN, MN0811CN‐BC4TN, M07‐292111, M07‐292111‐BC4TN1, and M07‐292111‐BC4TN1 was obtained and filtered using the same method/pipeline as described in the previous paragraph. These datasets were used to identify high‐quality SNPs between the triple‐mutant lines and their respective parent line or recurrent parent (WPT673‐7‐8‐8 and WPT673‐7‐12‐5 compared to Bert; MN0811CN‐BC4TN compared to MN0811CN; M07‐292111‐BC4TN1; and M07‐292111‐BC4TN1 compared to M07‐292111). To identify high‐quality SNPs, we filtered the bam file by mapping the quality of 30 using SAMtools (v 1.16.1) (Li et al., 2009) and then performing GATK haplotypecaller (v 4.1.2) (van der Auwera & O'Connor, 2020) on a comparison basis. To refine SNP calls, we applied GATK‐recommended filter (–filter‐expression “QD < 2.0 || FS > 60.0 || MQ < 45.0 || MQRankSum < −12.5 || ReadPosRankSum < −8.0” with additional “GQ < 25 and DP < 10” filter). To obtain the final dataset for SNP distribution analysis between each comparison, we removed variant sites that have missing data, heterozygous calls, as well as multiallelic sites. Data was visualized using R (v 4.3.4) using the circlize package (Gu et al., 2014).

### 2.5 Proteomics data‐independent acquisition (DIA)‐parallel accumulation–serial fragmentation (PASEF)

The total protein was extracted following the phenol extraction protocol as previously outlined (Mooney et al., 2004). Briefly, soybean seeds were ground and subjected to extraction using 2 mL of Tris pH 8.8 buffered phenol and 2 mL of extraction media (0.1 M Tris‐HCl pH 8.8, 10 mM EDTA, 0.9 M sucrose, 0.4% 2‐mercaptoethanol freshly added before use). The extraction mixture was vortexed for 30 s in a fume hood and agitated for 10 min at room temperature. After centrifugation for 10 min at 5000 g and 4°C, the phenol upper phase was transferred to a new microcentrifuge tube and re‐extracted with an equal volume of extraction buffer. The upper phase was collected, and five volumes of 0.1 M ammonium acetate in 100% methanol (stored at –20°C) were added. The mixture was vortexed and incubated at –20°C overnight. Precipitation was achieved by centrifugation at 16,000 g for 10 min at 4°C. The pellet was washed twice with 0.1 M ammonium acetate in methanol and twice with ice‐cold 80% acetone. The final protein pellets were suspended in 6 M urea, 2 M thiourea, and 100 mM ammonium bicarbonate. Protein concentration was determined using the Pierce 660 nm Protein Assay (Thermo Fisher Scientific) following the manufacturer's protocol.

For each sample, 30 µg of proteins were reduced with 10 mM dithiothreitol (DTT) at room temperature for 1 h and alkylated with 40 mM iodoacetamide (IAA) at room temperature for 1 h in the dark. Excess IAA was quenched by adding 10 mM DTT and incubating for 15 min. The urea buffer was diluted to 1 M urea with 50 mM ammonium bicarbonate. Sequential digestion with Trypsin (Thermo Fisher Scientific) was performed at a 1:100 enzyme‐to‐protein mass ratio, with the first digestion incubated at 37°C for 5 h and the second digestion incubated at 37°C overnight. Digested peptides were quenched with 0.5% TFA.

For spectral library generation, 5 µg of peptides from each sample set were pooled using the data‐dependent acquisition (DDA)‐PASEF acquisition method. Subsequently, 500 ng of peptides from each sample were loaded onto LCMS using the DIA‐PASEF acquisition method.

Proteome analyses were conducted using the EvoSep One liquid chromatography system, as previously described (Bache et al., 2018). Samples were analyzed with a 44‐min gradient for peptide elution (or Evosep One 30 SPD program). The chromatography setup included a 15 cm × 150 µm ID column packed with 1.5 µm C18 beads (Bruker PepSep) and a 20 µm ID zero dead volume electrospray emitter (Bruker Daltonik). Mobile phases A and B consisted of 0.1% formic acid (FA) in water and 0.1% FA in acetonitrile (ACN), respectively. The EvoSep One system was coupled online to a modified trapped ion mobility (IM) spectrometry quadrupole time‐of‐flight mass spectrometer (timsTOF Pro 2, Bruker Daltonik GmbH) via a nanoelectrospray ion source (Captive spray, Bruker Daltonik GmbH).

For DDA, default DDA settings were employed. For data‐independent acquisition (DIA), an in‐house optimized pyDIA‐PASEF method was utilized, featuring a variable window width over an m/z range of 300–1350 and an IM range of 0.65–1.45 Vs cm^−2^. The method incorporated two IM windows per DIA‐PASEF scan with variable isolation window widths adjusted based on precursor densities from the experimental spectral library. A total of 25 DIA‐PASEF scans were performed at a throughput of 30 SPDs, with a cycle time of 2.76 s.

The data analyses were conducted using Spectronaut version 17.0 with default settings, except for the proteotypicity filter, which was set to “Only protein group specific.” All data were searched against the Soybean_Glyma.Wm82.gnm4.ann1.T8TQ proteome using trypsin/LysC as digestion enzymes. Cysteine carbamidomethylation was set as a fixed modification, while methionine oxidation and acetylation at the N‐terminus were selected as variable modifications. A maximum of two missed cleavages and up to three variable modifications were allowed. To generate the spectral library for soybean seeds, all high‐pH (hph) fractions from the current project were combined with hph fractions from previous projects. In total, 41 runs were utilized, resulting in a library that comprised 5016 protein groups, 30,462 peptides, and 38,618 precursors. Protein quantification was performed using Spectronaut (version 17.0) with MS2‐level quantification based on area measurements. Precursor filtering was set to Identified (Qvalue), with Group Qvalue applied as the multi‐channel Qvalue filter. Cross‐run normalization was enabled, and the normalization strategy was set to local normalization. For protein grouping, major protein groups were defined by Protein Group ID, while minor grouping was based on stripped peptide sequences. Mean peptide quantity was used for major group quantification, and mean precursor quantity was used for minor group quantification. The top N peptides for both major and minor groups were selected with a maximum of 3 and a minimum of 1.

MS2‐level data for the samples were exported for differential protein expression analysis using the differentially expressed proteins (DEP) R/Bioconductor package (X. Zhang et al., 2018). Data from .csv files were then imported into a *SummarizedExperiment* object for analysis following the DEP workflow. Sample information was included in the annotation slot to store metadata. Before normalization, duplicate IDs were removed from the imported data. Data were normalized using the variance stabilizing normalization method, and proteins absent in >50% of the samples were removed. The remaining missing values were imputed using the *MinProb* method.

Differential protein expression was analyzed between “conventionally bred triple‐mutant lines vs. recurrent parents” and “CRISPR triple‐mutants vs. Bert,” using the recurrent parents and “wt‐Bert” as controls, with a significance threshold of 0.05. The DEP were then visualized using a volcano plot.

### 2.6 Trypsin inhibition assay

The trypsin inhibition assay was carried out as described by (Kim & Krishnan, 2023). Briefly, to prepare the sample, 20 mg of mature soybean seed powder was dissolved in 1 mL of a 10 mM NaOH solution and vigorously vortexed for 10 min at room temperature. The resulting mixture was then centrifuged at 16,000 g for 10 min. The clear supernatant obtained was subsequently used to measure the activities of trypsin inhibitors. For inhibition assay, 10 µL of seed extract was added to 190 µL of Assay buffer (50 mM Tris‐HCl, pH8.2 and 20 mM CaCl_2_). To this 400 µL of BAPNA solution (0.4 mg/mL) and 200 µL of trypsin (20 µg/mL) was added followed by incubation at 37°C for 10 min then reaction was stopped by adding 100 µL 30% acetic acid solution. Finally, absorbance was taken at 410 nm. Trypsin inhibitor units were calculated according to the previously outlined method. The results were expressed as the standard deviation from three independent replicates. An unpaired *T*‐test was used for computing the statistical significance among different genotypes.

### 2.7 Western blot analysis

The protein was extracted from seeds of each genotype using NaOH as previously described (Gillman et al., 2015). In short 20 ug of total seed protein from each genotype was resolved by Sodium Dodecyl Sulfate‐Polyacrylamide Gel Electrophoresis (SDS‐PAGE) and the protein samples were transferred to PVDF membrane (Thermo ScientificTM). The anti‐trypsin inhibitor antibody (ab34549; Abcam) was used as primary antibody, while Goat Anti‐Rabbit IgG H&L (horseradish peroxidase conjugated) (ab205718; Abcam) was used as secondary antibody. Protein bands were detected by chemiluminescence using ECLTM Prime Western Blotting reagents (AmershamTM, GE Healthcare). Coomassie blue stained membrane from the same blot was used to show the equal loading among all genotypes.

### 2.8 Protein digestibility assays

In vitro protein digestibility assays were performed using the Megazyme Protein Digestibility Assay Kit according to the manufacturer's instructions (Neogen).

### 2.9 Field‐based yield and maturity evaluations

The conventionally bred triple‐mutant MN0811CN‐BC4TN and CRISPR‐derived triple‐mutant lines WPT673‐7‐8‐8 and WPT673‐12‐5‐12 were grown in the field alongside their isogenic wild‐type plants (MN0811CN and Bert, respectively) in replicated field trials to assess their agronomic performance. The conventionally bred triple mutants M07‐292111‐BC4TN1 and M07‐292111‐BC4TN2 were also grown in these trials but were not included in downstream analyses because their isogenic recurrent parent M07‐292111 was not in these trials due to insufficient seed stocks.

The lines were evaluated in four field environments: SP2020 (St. Paul Campus Research Facilities, St. Paul, MN, 2020); RM2020 (Rosemount Research and Outreach Center, Rosemount, MN, 2020); RM2021 (Rosemount Research and Outreach Center, Rosemount, MN, 2021); and BE2021 (Sand Plain Research Farm, Becker, MN, 2021). In the SP2020 environment, lines were sown in 10‐feet‐long two‐row plots with 30″ row spacing following a randomized complete block design with three replications. In the other three environments, lines were planted in 12‐feet‐long two‐row plots with 30″ row spacing following a randomized complete block design with four replications. In all locations, seeds were sown at a planting density of 170,000 plants per acre (∼4 plants per square foot). Yield data were collected for all environments, while maturity date data (expressed in days after August 31) were collected in only three environments (BE2021, RM2020, and RM2021).

Yield and maturity data were analyzed using a restricted maximum likelihood mixed linear model implemented using JMP Pro v.16 (SAS Institute), with lines, environments, and line‐by‐environment interaction effects considered as fixed effects, and blocks nested within environments as random effects. Due to the differing number of replications across environments, least‐squares means were calculated for each line. Fisher's least significant difference was used to compare yield between triple‐mutant lines and their respective checks.

### 2.10 Grammar check

A grammar check was performed on the Abstract and Plain Language Summary sections using ChatGPT, OpenAI. Light editing was incorporated into these sections based on this grammar check.

## 3 RESULTS AND DISCUSSION

### 3.1 Development of anti‐nutritional triple‐mutant lines through conventional breeding

Previous work has described the identification and stacking of LE, KTi3, and P34 mutants (Bernard & Hymowitz, 1986; Bernard & Nelson, 1996; Herman & Burks, 2011; Joseph et al., 2006; Schmidt et al., 2015). The specific gene models harboring the mutants are identifiable for all three genes, including LE (gene model Glyma.02G012600), KTi3 (Glyma.08G341500), and P34 (Glyma.08G116300). The gene models listed above fit the naming convention of the Williams 82 annotations from reference genome versions a2, a4, and a6 (Espina et al., 2024). Schmidt et al. (2015), Song et al., 2016, and Valliyodan et al. (2019) combined/stacked the mutant alleles into a William 82 background and named the plant as *triple null*. We respectively backcrossed the three alleles into two different modern elite lines adapted to northern latitudes, M07‐292111 and MN0811CN. Molecular markers were used to track the allele introgression throughout four backcross generations, and then homozygous fixation of the mutant alleles through three generations of self‐pollination. This resulted in three superior introgressed lines, named as M07‐292111‐BC4TN1, M07‐292111‐BC4TN2 (both backcrossed into the M07‐292111 background), and MN0811CN‐BC4TN (backcrossed into the MN0811CN background).

### 3.2 Development of anti‐nutritional triple‐mutant lines through CRISPR‐based gene editing

CRISPR constructs with multiple gRNAs were designed to mutate the three genes in the background of the transformation‐friendly soybean cultivar Bert. The LE gene is located on chromosome 2, while the KTi3 and P34 genes are both located on chromosome 8. Based on the extensive distant locations between KTi3 (Chr08:48130204.48131130 forward, where Chr is chromosome) and P34 (Chr08:8979758.8982186 reverse) on chromosome 8, we assumed that mutant alleles of KTi3 and P34 would segregate independently in the progeny of a T_0_ transgenic plant, facilitating the stacking of mutant alleles in subsequent generations (genome coordinates shown are based on genome assembly Wm82.a6; Espina et al., 2024).

Three different transgenic cassettes were transformed into the Bert. Each of the three constructs contained unique gRNAs targeting each gene of interest (LE, KTi3, and P34). A detailed matrix of multiplex CRISPR targets is depicted in Figure S1. Genomic DNA extracted from the leaves of regenerated T_0_ plants was used for PCR screening of transgenes and target genes (primer sequences are shown in Table S3). From a total of 76 regenerated plants, 16 plants were PCR‐positive for the BAR amplicon and 14 plants were PCR‐positive for the GmUbi/AtCas9 amplicon (Figure S2).

Mutations of target genes in somatic tissues were revealed by comparing mobility of PCR products amplified from transgene‐positive plants and the wild‐type control. In addition, multiple somatic mutations were indicated by the formation of multiple bands of heteroduplex DNA in a PAGE gel (Figure S3). All three cassette versions showed evidence for targeted mutations (Figure S3; WPT673 and WPT681 were transformed with cassette I; WPT674 and WPT689 were transformed with cassette II; WPT675 was transformed with cassette III). Three T_0_ plants, WPT673‐7, WPT689‐12, and WPT674‐5, appeared to contain mutations in all three target genes, as multiple bands of unique mobility (shown in red rectangles of Figure S3) were detected in PCR amplified targets of the LE, KTi3, and P34 genes. In addition to heteroduplex DNA analysis, direct sequencing of PCR‐amplified targets after subcloning into pGEM‐T‐easy vector resulted in the identification of another T_0_ line, WPT673‐10, with mutations in all three target genes.

We sought to identify progeny plants carrying frameshift alleles in all three target genes while no longer harboring transgenic sequences. All 35 T_1_ plants from the WPT689‐12 lineage and all 75 T_1_ plants from the WPT673‐10 lineage were transgene‐positive. Furthermore, mutation profiles in the T_1_ progenies of WPT689‐12 exhibited only in‐frame mutations (3 bp and 12 bp deletions) in the target gene P34 (Figure S4). In contrast, ten out of 90 T_1_ plants in the WPT673‐7 lineage were transgene‐negative (Figure S5). In terms of mutation profile, the WPT673‐7 T_0_ plant carried mostly frameshift mutations for all three genes but also exhibited evidence for an in‐frame allele of P34: LE Δ4/Δ11; KTi3 Δ5/Δ12; P34 Δ3/Δ7 (Figure S4). Among the ten transgene‐negative T_1_ plants, only two plants, WPT673‐7‐8 and WPT673‐7‐12, exhibited frameshift mutations in both alleles of LE and KTi3. The P34 alleles were heterozygous for an in‐frame mutation (Δ3) and a frameshift mutation (Δ7) (Figure S6). Segregation in the T_2_ generation resulted in the recovery of two plants with homozygous frameshift mutations for all three genes: WPT673‐7‐8‐8 (Δ11/Δ11; KTi3 Δ5/Δ5; P34 Δ7/Δ7) and WPT673‐7‐12‐5 (Δ4/Δ4; KTi3 Δ5/Δ5; P34 Δ7/Δ7) (Figure S7).

### 3.3 Genomic analysis of the triple‐mutant lines

Whole genome sequencing (WGS) of the T_0_ plant WPT673‐7 identified transgene insertions on chromosomes 1 and 19. There was no evidence of transgene or vector backbone sequences in the WGS datasets of T_2_ plants WPT673‐7‐8‐8 and WPT673‐7‐12‐5, confirming the PCR evidence that the transgenes segregated out of these lineages.

**FIGURE 1:**
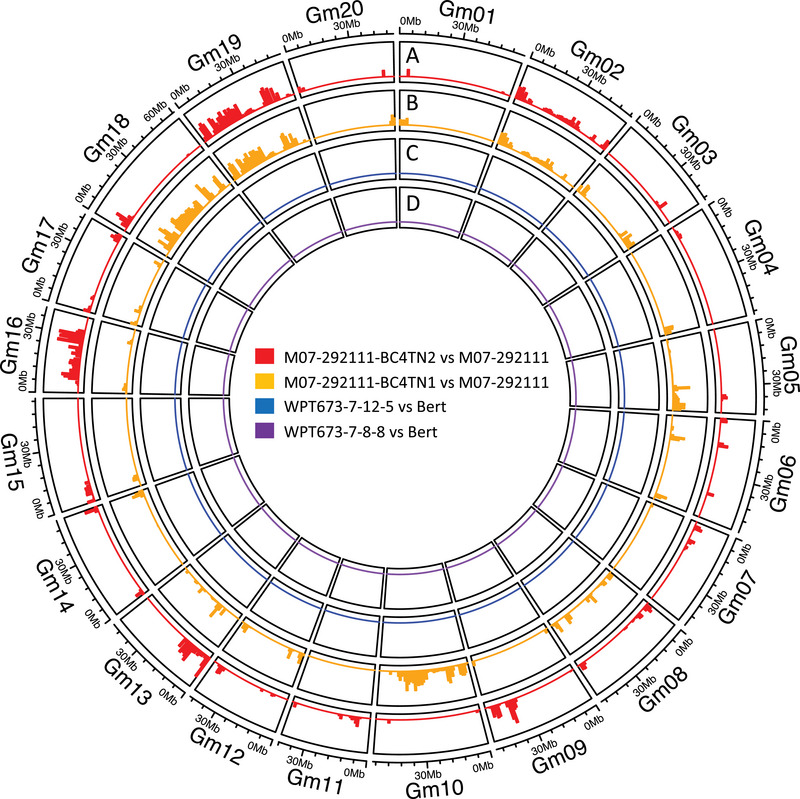
Genomic single‐nucleotide polymorphism (SNP) variation between conventionally bred triple‐mutant lines and their recurrent parents (A–B) and the clustered regularly interspaced short palindromic repeats (CRISPR)‐derived triple‐mutant lines and their parental line Bert (C–D). The colored tracks represent the abundance of homozygous SNPs found within 1 MB intervals across each chromosome for each comparison. Each circle represents one comparison across all 20 chromosomes (Gm01‐Gm20 [where Gm is glycine max chromosome]). (A) Red tracks show M07‐292111‐BC4TN2 compared to M07‐292111; (B) Orange tracks show M07‐292111‐BC4TN1 compared to M07‐292111; (C) Blue tracks show WPT673‐7‐12‐5 (where WPT is whole plant transformation) compared to Bert; (D) Purple tracks show WPT673‐7‐8‐8 compared to Bert.

We compared the relative haplotype and SNP variation between the conventionally bred triple‐mutants and the CRISPR‐derived triple‐mutants to their respective control lines. We found that M07‐292111‐BC4TN1 and M07‐292111‐BC4TN2 each had over 400,000 SNPs when compared to their recurrent parent M07‐292111 (Table 1). The vast majority of these SNPs were concentrated within clear linkage blocks (Figure 1A,B), particularly on chromosomes 16, 18, and 19. There was also clear evidence of SNPs clustered at the loci surrounding the three introgressed mutant genes (at positions ∼1.1 Mb on chromosome 2, and at positions ∼9.0 Mb and ∼48.1 Mb on chromosome 8) in both M07‐292111‐BC4TN1 and M07‐292111‐BC4TN2 (Figure 1A,B). These findings matched expectations, as backcross breeding will introgress the mutation(s) of interest along with DNA linked to the mutation(s), in addition to stochastic DNA introgressions elsewhere in the genome. This is discussed in greater detail in Section 3.7.

**TABLE 1:** Abundance of homozygous single‐nucleotide polymorphisms (SNPs) identified in comparisons of conventionally bred triple‐mutant lines to their recurrent parents and the clustered regularly interspaced short palindromic repeats (CRISPR)‐derived triple‐mutant lines to the parental line Bert.

Breeding method	Genotype comparison	No. of SNPs
Conventionally bred	M07‐292111‐BC4TN1 vs. M07‐292111	517,019
Conventionally bred	M07‐292111‐BC4TN2 vs. M07‐292111	429,618
CRISPR‐derived	WPT673‐7‐8‐8 vs. Bert	114
CRISPR‐derived	WPT673‐7‐12‐5 vs. Bert	106

The CRISPR‐derived triple‐mutants each had slightly greater than 100 total SNPs compared to their parental line Bert (Table 1). This SNP abundance was scattered throughout the genome and was too low to register a visible cluster in any one location (Figure 1C,D). The comparison between WPT673‐7‐12‐5 and Bert exhibited three missense mutations within annotated genes, predicting amino acid changes in Glyma.02G012600 (T in Bert/S in WPT673‐7‐12‐5), Glyma.12G172250 (L/F), and Glyma.07G121900 (S/A). The comparison between WPT673‐7‐8‐8 and Bert exhibited two missense mutations within annotated genes, predicting amino acid changes in Glyma.08G279200 (S in Bert/C in WPT673‐7‐12‐5) and Glyma.07G121900 (S/A). Both WPT673‐7‐12‐5 and WPT673‐7‐8‐8 shared the same polymorphism for Glyma.07G121900, which may be expected since the lines are derived from the same T_0_ event. None of the missense mutations described above result in stop codons or other obviously deleterious effects, though the S/A and S/C substitutions do have different polarity. It is also worth noting that the stock of Bert seeds used in these experiments has been maintained via bulk‐harvesting for several generations. It is thus assumed that some SNPs have differentially accumulated in the stock prior to the transformation and editing process, resulting in baseline SNP differences between the plant selected as the Bert control and the plant subjected to the transformation pipeline. Therefore, it is unclear what portion of the SNPs can be attributed to natural processes, as opposed to the tissue culture, transformation, and CRISPR‐editing procedures.

### 3.4 Seed protein expression of the triple‐mutant alleles

**FIGURE 2:**
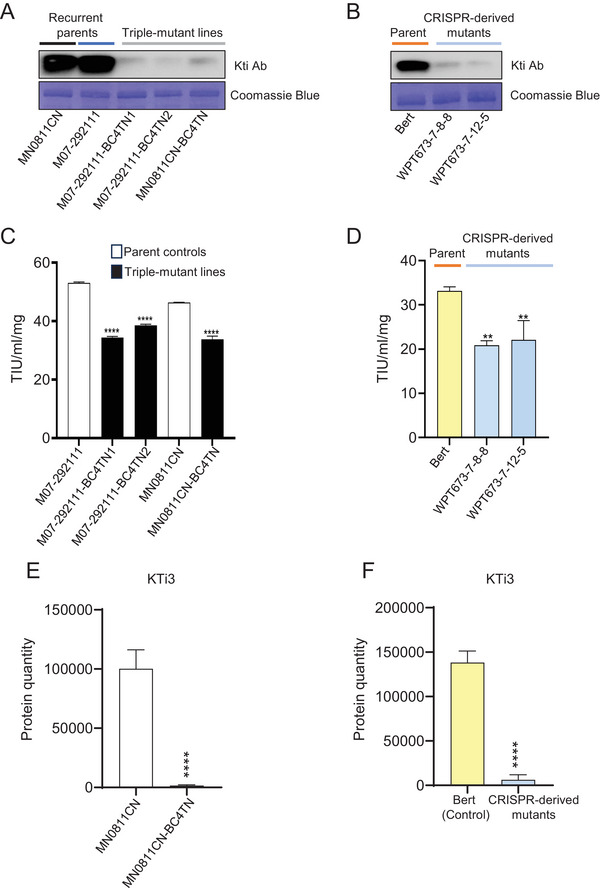
Evidence for knockout of the Kunitz trypsin inhibitor protein. (A–B) Confirmation of Kunitz trypsin inhibitor protein reduction by Western blot analysis using a Kunitz trypsin inhibitor (KTI) antibody in conventionally bred triple‐mutant lines (A) and clustered regularly interspaced short palindromic repeats (CRISPR)‐derived triple‐mutant lines (B), respectively. MN0811CN‐BC4TN is derived from MN0811CN; M07‐292111‐BC4TN2 and M07‐292111‐BC4TN1 are derived from M07‐292111. WPT673‐7‐12‐5 and WPT673‐7‐8‐8 (where WPT is whole plant transformation) are edited lines developed in the Bert background. (C–D) Trypsin inhibition activity in conventionally bred triple‐mutant lines (C) and CRISPR‐derived triple‐mutant lines (D), respectively. Black asterisks in (C) correspond to recurrent parent MN0811CN, while blue asterisks correspond to recurrent parent M07‐292111. Shown is the average of three technical replicates, and error bars indicate SD. **p < 0.001, ***p < 0.001, ****p < 0.0001, adjusted *p*‐value for one‐way analysis of variance (ANOVA) (C–D). (E–F) Confirmation of KTi3 protein reduction in total seed proteome by dia‐parallel accumulation–serial fragmentation (PASEF)‐based proteomics analysis. (E) Each genotype value represents the average of two technical replicates of ∼100 bulk seeds from 20–25 field‐harvested plants per genotype. (F) Each genotype value represents the average of three technical replicates of ∼100 bulk seeds from 20–25 field‐harvested plants per genotype. Error bars indicate SD. **p < 0.001, ***p < 0.001, ****p < 0.0001, adjusted *p*‐value Tukey *t*‐test.

To analyze the seed protein profiles, we conducted a series of experiments on extracted proteins from seeds at the R8 developmental stage (mature seeds) grown in fields at St. Paul, MN, in 2021 and 2022. First, we analyzed the abundance of KTi3 proteins in both the conventionally bred and CRISPR‐derived lines. Western blot and SDS‐PAGE analyses indicated that the parental control lines (M07‐292111, MN0811CN, and Bert) each exhibited strong signals for the KTi proteins, whereas all triple‐mutant lines (M07‐292111‐BC4TN1, M07‐292111‐BC4TN2, MN0811CN‐BC4TN, WPT673‐7‐8‐8, and WPT673‐7‐12‐5) exhibited severe reductions in the KTi protein signal (Figure 2A,B; Figures S8). The Western blot analysis also included a triple‐mutant control in the Williams 82 background (labeled as Williams82TN in Figure S8), which has previously been characterized (Schmidt et al., 2015). This outcome was further confirmed by DIA parallel accumulation–serial fragmentation (PASEF)‐based whole seed proteomics study of these proteins (Figure 2E,F). KTi enzymatic activity was also reduced in the triple‐mutant lines (Figure 2C,D); however, not as severely as might be expected based on the reduction in the abundance of protein. The conventionally bred and CRISPR lines showed proportionally similar outcomes in terms of reduction in KTi protein abundance and reduction in KTi enzymatic activity.

**FIGURE 3:**
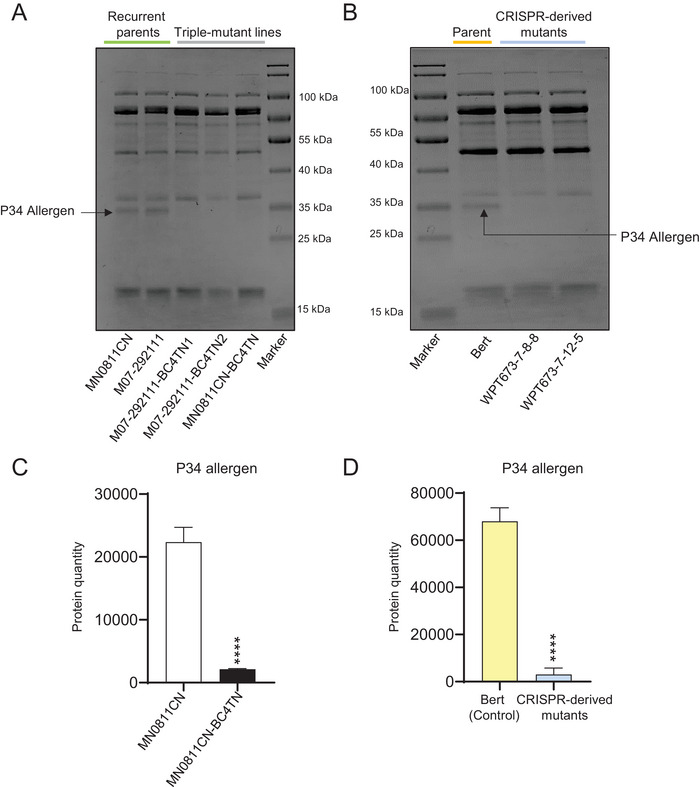
Evidence for knockout of the P34 protein. (A–B) Confirmation of P34 allergen protein reduction using SDS‐PAGE in conventionally bred triple‐mutant lines (A) and clustered regularly interspaced short palindromic repeats (CRISPR)‐derived triple‐mutant lines (B), respectively. (C–D) Confirmation of P34 allergen protein reduction in total seed proteome by dia‐parallel accumulation–serial fragmentation (PASEF) based proteomics analysis. Sampling, assays, and statistical tests for (C–D) were performed as described in the Figure 2 (E–F) legend. Error bars indicate SD. ****p < 0.0001, adjusted *p*‐value Tukey *t*‐test.

The abundance and migration pattern of the protease‐like allergen P34 protein allow it to be visualized in a standard SDS‐PAGE protein electrophoresis gel. Figure 3A,B shows that the P34 protein bands are visible in the control lines, but not visible in the triple‐mutant lines, both for the conventionally bred and CRISPR‐derived materials. This finding is confirmed by diaPASEF‐based proteomics, showing a nearly complete reduction of P34 counts in the triple‐mutant lines (Figure 3C,D).

**FIGURE 4:**
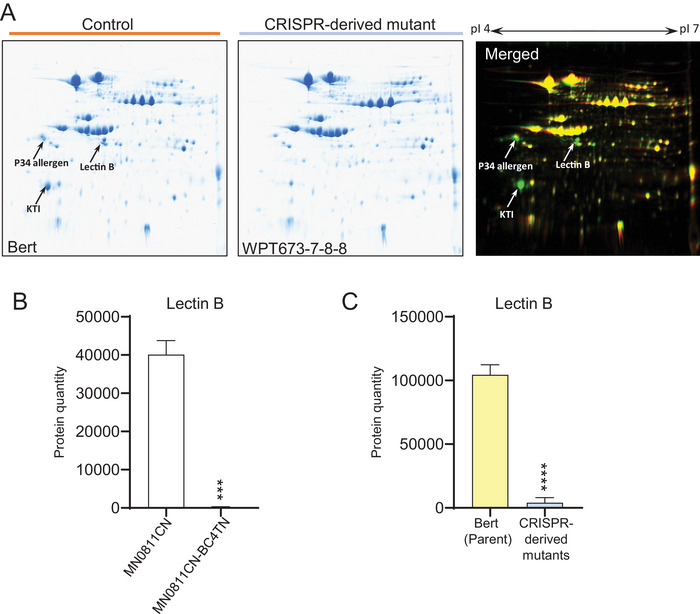
Evidence for knockout of the triple‐mutant proteins. (A) Confirmation of lectin B, Kunitz trypsin inhibitor (KTI), and P34 allergen deletion by 2D‐PAGE analysis in the clustered regularly interspaced short palindromic repeats (CRISPR)‐derived triple‐mutant line WPT673‐7‐8‐8 (where WPT is whole plant transformation) compared to Bert. Overlay of two separate two‐dimensional gels of soybean seed proteins using Delta2D software. Gels were scanned, and resulting images were assigned two different colors (green = Bert; red = WPT673‐7‐8‐8) to visualize their differences. Yellow demonstrates similar protein quantities in each. Green spots (arrows) indicate proteins present in Bert while absent in the triple mutant. (B–C) Confirmation of Lectin B protein reduction in total seed proteome by dia‐parallel accumulation–serial fragmentation (PASEF)‐based proteomics analysis. Sampling, assays, and statistical tests for (B–C) were performed as described in Figure 2E,F legend. Error bars indicate SD. ***p < 0.001. ****p < 0.0001, adjusted *p*‐value Tukey *t*‐test.

The diaPASEF‐based proteomics analysis also indicated a nearly complete loss of the LE protein in the triple‐mutant lines compared to the control lines (Figure 4C,D). Proteomic analysis using 2D gels (Figure 4A,B; Figure S9) further confirmed these findings, as the signals from KTi3, P34, and LE were clearly visible in the control plants and not visible in the triple‐mutant lines. Additional analyses of KTi3, P34, and LE protein reduction are provided for the conventional and CRISPR triple‐mutant plants in Figures S10 and S11, respectively. Figure S12 shows the global differential protein expression patterns for the conventionally bred and CRISPR‐derived lines compared to their respective controls. In both cases, the protein products from the three mutated genes are clear outliers compared to the rest of the proteome.

### 3.5 Seed proteome rebalancing and protein digestibility in the triple‐mutant lines

**FIGURE 5:**
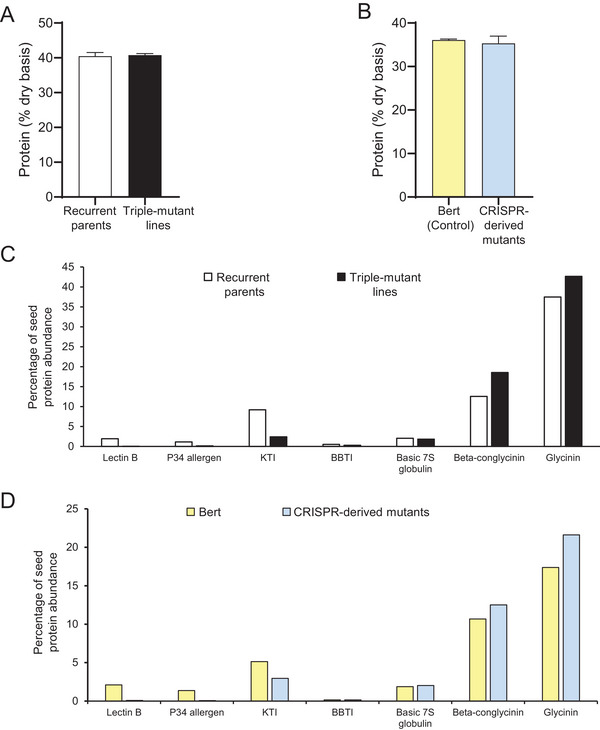
Protein rebalancing in triple‐mutant lines. (A) Total protein content of recurrent parental lines and conventionally bred triple‐mutant lines based on near‐infrared reflectance (NIR). “Recurrent parents” indicates the combined values of MN0811CN and M07‐292111. “Triple‐mutant lines” indicates the combined values of MN0811CN‐BC4TN, M07‐292111‐BC4TN1, and M07‐292111‐BC4TN2. (B) Total protein content of Bert (parent) and clustered regularly interspaced short palindromic repeats (CRISPR)‐derived triple‐mutant lines. “CRISPR‐derived lines” indicates the combined values of WPT673‐7‐8‐8 and WPT673‐7‐12‐5 (where WPT is whole plant transformation). Shown is the average of a minimum of three biological replicates of each genotype, and error bars indicate SD. An unpaired *t*‐test showed no significant difference (both for A–B). (C–D) Protein rebalancing by other storage proteins in conventionally bred triple‐mutant lines (C) and CRISPR‐derived triple‐mutant lines (D) compared to their respective controls. Percentage of each protein was calculated from the whole seed proteome by dia‐parallel accumulation–serial fragmentation (PASEF)‐based proteomics. Sampling, assays, and statistical tests for (C–D) were performed as described in the Figure 2E,F legend.

Despite the reduction/elimination of three highly abundant seed proteins in the triple‐mutant lines, the estimate of total seed protein, as measured by near‐infrared reflectance, showed no statistical difference in the comparisons of the triple‐mutants to their respective parental lines (Figure 5A,B). diaPASEF‐based proteomics indicated that storage protein rebalancing, particularly upregulation of the glycinin and beta‐conglycinin proteins (Figure 5C,D; Tables S4 and S5), may be largely responsible for the unaltered level of total seed protein in these comparisons. Evidence for this type of soybean seed storage protein rebalancing has been previously reported in the literature (Gillman et al., 2015).

Given the reduction of trypsin activity and seed allergens, and the development of new alleles for these traits in the CRISPR mutants, we performed an in vitro test of protein digestibility in the triple‐mutant lines (Figure S13), which simulates gastrointestinal protein digestion by treatment with pepsin, followed by treatment with trypsin and chymotrypsin. Undigested proteins are precipitated, and amino acids released by the digestion are quantitated. There was no evidence that the mutant lines have improved protein digestibility compared to the respective control lines (Figure S13). Given that the KTis contribute a large portion of protease inhibitor activity in soybeans (Liu, 2024) and KTi3 is the most highly expressed KTi in soybean seeds (Wang et al., 2023), this is a somewhat unexpected result. It is possible that the level of reduction in total KTi protein product (Figure 5C,D) was not sufficient to alter digestibility, or other seed proteins are compensating for the KTi3 mutation, such as Bowman‐Birk inhibitors (see previous work by Gillman et al., 2015).

### 3.6 Harvestable yield is unchanged in the anti‐nutritional triple‐mutant lines

**FIGURE 6:**
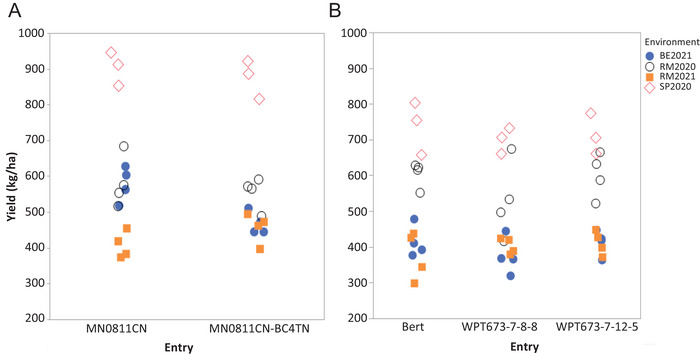
Field‐based seed yield results for triple‐mutant lines and parental control lines in four environments (year by location). (A) Yield of the conventionally bred MN0811CN‐BC4TN compared to its parent MN0811CN. (B) Yield of the clustered regularly interspaced short palindromic repeats (CRISPR)‐edited lines WPT673‐7‐8‐8 and WPT673‐7‐12‐5 (where WPT is whole plant transformation) compared to the parent Bert. Each symbol indicates a replicate per year per location. Year by locations include Becker, MN in 2021 (BE2021); Saint Paul, MN, in 2020 (SP2020); and Rosemount, MN, in 2020 (RM2020) and 2021 (RM2021).

The conventionally bred and CRISPR triple‐mutant lines were compared to their respective control lines for harvestable soybean yield across four environments (year by location). Figure 6 shows the yield results for each of the eight lines in all four environments. When comparing adjusted means for yield across all environments, no significant differences were observed between triple‐mutant lines and their respective parental lines (Tables S6 and S7). Significant differences (*p* < 0.05) were observed between lines, which is driven by differences among the background genotypes (M07‐292111, MN0811CN, and Bert) (Table S8). Highly significant differences (*p* < 0.001) were observed between environments and environment by line interactions (Table S8). The majority of variation for yield was due to differences between environments (Table S8) and can be attributed to differences in soil fertility between environments and precipitation across years. Significant differences were observed in maturity (days after August 31) between all triple‐mutant lines and their respective checks, though these differences were small for the CRISPR‐derived lines WPT673‐7‐8‐8 (20.5 days), WPT673‐7‐12‐5 (20.3 days) versus Bert (18.8 days), and for the conventionally bred line MN0811CN‐BC4TN (6.2 days) compared to its control MN0811CN (7.6 days).

### 3.7 Regulatory implications

The question of risk is fundamental to understanding and determining how a given technology may be regulated. Modern datasets indicate that crop germplasm harbors much greater natural genomic variation than might have been presumed prior to the advent of the high‐throughput genome assembly and resequencing era. This poses important considerations from a regulatory standpoint, as one may reconsider the threshold of what constitutes risk derived de novo from biotechnology compared to the tremendous variation extant in the standing and evolving germplasm (Graham et al., 2020).

Within this context, the present study offers relevant comparisons at two different levels: (1) The comparison of triple‐mutant lines to their respective parental lines per se; (2) the comparison of comparisons—how the responses of the conventional and CRISPR triple‐mutants compare to one another. In other words, we expect that mutating major seed proteins will cause differences between the triple‐mutant and their respective parents; however, the key question from a regulatory standpoint may be whether the types of changes we observe in the CRISPR‐derived lines are different than the types of changes we observe in the conventionally bred lines.

A summary of our findings indicates that the conventionally bred and CRISPR‐derived lines behave similarly with respect to their parental lines in almost every measurable way. This includes the following: (1) Both conventional and CRISPR lines show similar changes compared to their respective parents in terms of loss/elimination of the three target proteins; (2) Both conventional and CRISPR lines show similar changes compared to their respective parents in terms of proteome rebalancing of major storage proteins in the seed; (3) Both conventional and CRISPR lines show similar changes compared to their respective parents in terms of trypsin inhibitor enzymatic activity; (4) Both conventional and CRISPR lines show no statistical differences compared to their respective parents in terms of total seed protein content; (5) Both conventional and CRISPR lines show no statistical differences compared to their respective parents in terms of protein digestibility; (6) Both conventional and CRISPR lines show no statistical differences compared to their respective parents in terms of harvestable yield.

There is only one clear difference between the conventionally bred and CRISPR‐derived lines compared to their respective recurrent/background parent lines: There is greater sequence variation between the conventional lines and their recurrent parents compared to the variation between the CRISPR‐derived lines and Bert. This result fits expectations, as backcross breeding was effective at introgressing the three mutations of interest but also necessarily introduced segments of DNA linked to the mutations, in addition to stochastic DNA introgressions elsewhere in the genome. Furthermore, the varieties used in this study were originally developed from F_4_‐derived breeding populations; thus, some of the haplotype variation in the present comparisons is due to remnant heterogeneity between the plant used for backcrossing versus the plants genotyped as representing the recurrent parent.

The SNP variation exhibited between the conventionally bred lines and their recurrent parent is not necessarily an undesirable outcome. The genomic identity of a recurrent parent and a conventionally bred introgression line is a function of the number of backcrosses performed. The goal of backcross breeding is not to produce genetically identical lines, but rather to produce a line that is functionally similar to the recurrent parent while adding/introgressing favorable trait(s) from the donor line. If a breeder so desired, one could execute a sufficient number of backcrosses to generate an introgression line that is essentially identical to the recurrent parent, while adding the intentionally introgressed alleles. This outcome would be analogous to the CRISPR‐derived product wherein the parental line and the CRISPR‐derived line carry almost no genomic variation besides the targeted genes of interest.

We did not see evidence of significant off‐targeting or other DNA sequencing variants introduced by the gene editing process, beyond a small subset of sequence variants that may have been generated by natural mutation processes across generations. However, the editing events described in this work were performed in the background line Bert, which is not a modern‐day elite cultivar. Thus, deployment of these edited alleles would require multiple generations of backcrossing into elite genetic backgrounds. This limitation may be overcome with advances in genotype‐independent transformation/editing of soybean, which is an area of active research (e.g., Alok et al., 2025; Kshetry et al., 2025; Zhong et al., 2024). This would enable direct gene editing within elite genetic backgrounds.

Mutation breeding is a third method that could be used to develop triple‐mutant lines analogous to those described in this study. The mutation breeding process confers certain advantages, as it is exempt from regulation in most parts of the world. However, there is no basis to hypothesize that mutation breeding would be more efficient or produce genetically superior products to those described here. Perhaps most importantly, the process of developing mutant populations using unregulated treatments (e.g., chemical or irradiation treatments) necessarily generates numerous background mutations. Most induced mutations would have minimal or no effect on the plant phenotype, but some would have a negative effect on phenotype. Therefore, there is no obvious advantage to mutation breeding in cases where desired knockout alleles already exist in the current germplasm.

Transgenic gene silencing offers another alternative strategy for developing lines analogous to those described in this study. This approach typically involves inserting a transgene into the plant genome that is capable of triggering downstream silencing of target genes through RNA interference (RNAi) processes (Saurabh et al., 2014). From a plant breeding perspective, this system offers clear advantages. First, it is possible to use a single transgene to silence multiple genes, such as the three genes described in this study. Thus, breeders can select the trait(s) of interest through a single locus, rather than selecting a “stack” of unlinked loci. Furthermore, the RNAi transgene can be controlled by a promoter of choice, such that silencing of target genes can be specified in a developmenta‐ or tissue‐specific condition. This is particularly advantageous when gene expression is required in certain tissues while preferably silenced in other tissues (such as seeds, as in the present study). The main disadvantage of RNAi is that it typically requires the use of a regulated transgenic event.

The possibility of using different methodologies to develop analogous products is exemplified by the various paths that have been used to develop high oleic soybeans. This includes products developed by backcross introgression of natural alleles (Pham et al., 2010), mutation breeding (Sweeney et al., 2017), gene editing (Haun et al., 2014), and transgenic RNAi (Brink et al., 2014; Buhr et al., 2002; Knowlton, 1999; Mroczka et al., 2010). While these methods are differentially regulated and each have their distinct advantages and disadvantages, they produced functionally analogous products. This example illustrates the peculiarity of the current regulatory environment, which focuses on the process of trait development rather than the product/trait per se.

In summary, there is no evidence that the multiplex CRISPR editing used in this study introduced different or more unexpected outcomes than did the conventional backcross approach. Furthermore, the CRISPR triple‐mutant was more similar to its parental line than the conventionally bred lines were to their recurrent parents. Based on these findings, we see no scientific basis to support the notion that multiplex gene editing is more unpredictable or inherently risky compared to the conventional breeding strategy of introgressing standing mutations, which has been safely used for several decades in breeding programs.

## AUTHOR CONTRIBUTIONS

**Junqi Liu**: Data curation; investigation; methodology; validation; visualization; writing—original draft. **Ritesh Kumar**: Data curation; investigation; methodology; validation; visualization; writing—original draft. **Samatha Gunapati**: Data curation; investigation; methodology; validation; writing—original draft. **Steven Mulkey**: Formal analysis; investigation; methodology; visualization; writing—original draft. **Yinjie Qiu**: Formal analysis; visualization. **Yer Xiong**: Investigation. **Vishnu Ramasubramanian**: Formal analysis; visualization. **Jean‐Michel Michno**: Formal analysis. **Praveen Awasthi**: Methodology; visualization. **Daniel D. Gallaher**: Investigation. **Thi Thao Nguyen**: Formal analysis. **Won‐Seok Kim**: Investigation; visualization. **Hari B. Krishnan**: Investigation; visualization. **Aaron J. Lorenz**: Conceptualization; funding acquisition; methodology; supervision. **Robert M. Stupar**: Conceptualization; funding acquisition; supervision; visualization; writing ‐ original draft; writing ‐ review and editing

## CONFLICT OF INTEREST STATEMENT

Junqi Liu and Robert M. Stupar are co‐inventors on a patent concerning plant gene editing. R.M.S is a co‐inventor on a patent related to transformation efficiency of legume crop species. R.K. and Y.X. are currently employed by companies with research and development efforts in the plant biotechnology space. The remaining authors declare no competing interests.

## Supporting information




**Figure S1. Schematic representation of T‐DNA regions of the CRISPR constructs used in this study**. (A) Diagram of the transgene components in three constructs. They all include a Cas9 gene fused to Csy4, a TREX2 enzyme, a BAR selectable marker, and three gRNA sequences targeting the respective three genes (labeled as LE, P34, and KTi3 here). Note that the position of the three gRNAs is shifted among the three constructs. (B) The gRNA sequences used in each of the three constructs.
**Figure S2: PCR amplification of transgenes from regenerated soybean plants**. Genomic DNA was extracted from young leaves of regenerated putative T_0_ soybean plants. PCR amplification of transgenes was performed with GmUbi/Cas9 primers spanning the junction between GmUbi promoter and AtCas9 coding region. CNT: Control non‐transgenic soybean plant. H_2_O: water as a negative PCR control. The vertical white bar in the upper image indicates where images were spliced together to simplify the visual comparison of samples.
**Figure S3. Preliminary screening of somatic mutations in the primary transformants (T_0_)**. PCR amplified target regions from the LE, KTi3, and P34 genes were resolved in gradient 4–20% (1 x TBE) polyacrylamide gels. PCR amplicons with heteroduplex DNA of three target genes were displayed for LE (A), KTi3 (B), and P34 (C), respectively. The WPT pre‐fix was left off the lane labels for simplicity of viewing. The vertical white bars dividing lanes 688‐15 and 673–7 in parts (B) and (C) indicate where images were spliced together to simplify the visual comparison of samples. Red rectangles indicate the presence of mutations, as altered mobility is presumably caused by heteroduplex formation in the annealed PCR product. The T0 plants (WPT673‐7, WPT689‐12, and WPT674‐5) appeared to contain mutations in all three target genes. M: Molecular weight size marker.
**Figure S4. Heritable mutations transmitted from the T_0_ to the T_1_ generation**. Note that WPT689‐12 exhibited only in‐frame mutations (3 bp and 12 bp deletions) in the P34 target. Meanwhile, the WPT673‐7 progeny showed evidence for inheritance of frameshift alleles for all three target genes.
**Figure S5. Segregation of transgenes in T_1_ progenies of event WPT673‐7**. Genomic DNA was extracted from young leaves of T_1_ plants derived from WPT673‐7. PCR amplification of transgenes was performed with GmUbi/Cas9 primers spanning the junction between GmUbi promoter and AtCas9 coding region. Sample numbers were serial numbers of T_1_ plants preceded with WPT673‐7‐. Ten out of ninety T_1_ plants were transgene‐negative: WPT 673‐7‐2, 673‐7‐8, 673‐7‐9, 673‐7‐12, 673‐7‐13, 673‐7‐15, 673‐7‐38, 673‐7‐46, 673‐7‐83 and 673‐7‐89; H_2_O: water as negative PCR control; M: Molecular weight size marker. The vertical white bars indicate where gel images were spliced together to simplify the comparison of samples.
**Figure S6. Selection of T_2_ progenies harboring homozygous mutations with potential knock‐out effects for all three target genes**. The presence of transgene sequences were screened using PCR and whole‐genome sequencing in the T_0_ and T_1_ plants. The non‐transgenic status of the two T_2_ plants was confirmed with PCR and whole‐genome sequencing.
**Figure S7. Mutation profiles of LE, KTi3 and P34 genes in the T_2_ lines used for protein/proteomic analysis in this study**. Both of these plants were non‐transgenic and carried homozygous frameshift alleles for all three target genes.
**Figure S8. Detection of KTi‐1 and KTi‐3 proteins in soybean seeds for conventionally‐bred triple‐mutants compared to recurrent parents**. (A‐B) Seed proteins remaining in the supernatant after fractionation with 100 mM calcium chloride were resolved by 13.5% SDS‐PAGE. (C‐D) Isopropanol extracted seed proteins were resolved by 13.5% SDS‐PAGE. Panel A, C: Coomassie Blue stained gel. Panel B, D: Immunoblot analysis with soybean KTi antibodies. Marker = Protein Molecular weight markers.
**Figure S9. Two‐dimensional gel electrophoresis of soybean seed proteins in WPT673‐7‐12‐5 compared to Bert**. Seed proteins (300 µg) were separated by isoelectric focusing on pI 4–7 strips, followed by SDS‐PAGE on 10–16% gradient gels. Following electrophoresis, the gels were stained with Colloidal Coomassie Blue G‐250. The position and sizes of the protein molecular weight markers in kDa are shown on the left side of the figure. (A) Bert; (B) WPT673‐7‐12‐5. (C) Overlay of two separate two‐dimensional gels of soybean seed proteins using Delta2D software. Gels were scanned and resulting images were assigned two different colors (green = Bert; red = WPT673‐7‐12‐5) to visualize the differences between the two. Yellow demonstrates similar protein quantities in each. Green color demonstrates absence of that protein species in the WPT673‐7‐12‐5 mutant. Arrows point to proteins missing in WPT673‐7‐12‐5.
**Figure S10: SDS‐PAGE analysis of ethanol‐extracted soybean seed proteins for conventionally‐bred triple‐mutants compared to recurrent parents**. Proteins were separated with 15% SDS‐PAGE and visualized by staining the gel with Coomassie Blue. M = Protein Molecular weight markers. The * symbol indicates the location of P34 and lectin proteins.
**Figure S11. Detection of KTi‐1, KTi‐3, P34 and lectin proteins in soybean seeds across biological replicates comparing the CRISPR‐derived triple‐mutant and Bert**. (A‐B) Seed proteins remaining in the supernatant after fractionation with 100 mM calcium chloride were resolved by 13.5% SDS‐PAGE. (A) Coomassie Blue stained gel. (B) Immunoblot analysis with soybean KTi antibodies. The position and sizes of the protein molecular weight markers in kDa are shown on the left side of the figure. (C) SDS‐PAGE analysis of ethanol‐extracted soybean seed proteins. Proteins were separated with 15% SDS‐PAGE and visualized by staining the gel with Coomassie Blue. The position and sizes of the protein molecular weight markers in kDa are shown on the left side of the figure. The * symbol indicates the location of P34 and lectin proteins.
**Figure S12. Differentially expressed proteins (DEP)**. (A) DEPs in conventionally‐bred triple‐mutant lines compared to recurrent parents. (B) DEPs in CRISPR‐derived triple‐mutants compared to Bert. Blue dots represent downregulated proteins, while red dots represent upregulated proteins. Fold change is shown as log_2_ values.
**Figure S13. Protein digestibility was not affected in triple‐mutants**. Protein digestibility in conventionally‐bred triple‐mutant lines (A) and CRISPR‐derived triple‐mutant lines (B). MN0811CN, M07‐292111 (recurrent parent controls), M07‐292111‐BC4TN1, M07‐292111‐BC4TN2, and MN0811CN‐BC4TN (three conventionally‐bred triple‐mutants), Bert (Control), WPT673‐7‐8‐8 and WPT673‐7‐12‐5 (two CRISPR‐derived triple‐mutants in the Bert background). Each genotype value represents the average of three technical replicates of ∼100 bulk seeds from 20–25 field‐harvested plants per genotype. Error bars indicate SD. One‐way analysis of variance analysis showed no significant difference among different genotypes.

**Table S1**. Oligos for cloning 3plex gRNAs
**Table S2**. Assembly of gRNA spacers by PCR amplification
**Table S3**. Oligos for amplifying transgenes to detect transgenic plants; oligos for amplifying target genes and/or for CAPS PCR
**Table S6**. Least squared means for yield and maturity date for five triple‐knockout lines and three checks grown in four Minnesota environments.
**Table S7**. Comparison of least square (LS) means of yield (bushels/acre) for eight triple‐knockout lines (above) grown in four Minnesota environments (below) using Tukey's Honestly Significant Differences (HSD).^1^
**Table S8**. Analysis of Variance for the components of field‐based yield trials for five triple‐knockout mutant lines and three parental lines grown in four Minnesota environments (year by location).

Table S4. diaPASEF based proteomics comparing the conventionally‐bred triple mutant MN0811CN‐BC4TN with its parent line MN0811CN.

Table S5. diaPASEF based proteomics comparing the CRISPR triple mutants WPT673‐7‐8‐8 and WPT673‐7‐12‐5 with their parent line Bert.

## Data Availability

Sequencing data for the samples in this study are deposited in the Sequence Read Archive (http://www.ncbi.nlm.nih.gov/sra/) under accession number PRJNA1214329. The workflow for SNP filtering is available on GitHub (https://github.com/qiuxx221/stuparr_8_SNP_profile_project). The workflow for differential protein expression analysis is available (https://github.com/UMN‐Lorenz‐Group/stupar_triple_null_dep.git).

## References

[tpg270056-bib-0001] Alok, A. , Raman, V. , D'Agostino, L. , Kshetry, A. O. , Rai, K. M. , Wang, C. , Gunapati, S. , Stupar, R. M. , Patil, G. B. , & Zhang, F. (2025). Developmental regulators enable rapid and efficient soybean transformation and CRISPR‐mediated genome editing. bioRxiv. 10.1101/2025.03.03.641322 PMC13016980 41370232

[tpg270056-bib-0002] Bache, N. , Geyer, P. E. , Bekker‐Jensen, D. B. , Hoerning, O. , Falkenby, L. , Treit, P. V. , Doll, S. , Paron, I. , Müller, J. B. , Meier, F. , Olsen, J. V. , Vorm, O. , & Mann, M. (2018). A novel LC system embeds analytes in pre‐formed gradients for rapid, ultra‐robust proteomics. Molecular & Cellular Proteomics, 17, 2284–2296. 10.1074/mcp.TIR118.000853 30104208 PMC6210218

[tpg270056-bib-0003] Bernard, R. L. , & Hymowitz, T. (1986). Registration of L81‐4590, L81‐4871, and L83‐4387 soybean germplasm lines lacking the Kunitz trypsin inhibitor. Crop Science, 26, 650–651. 10.2135/cropsci1986.0011183x002600030058x

[tpg270056-bib-0004] Bernard, R. L. , & Nelson, R. L. (1996). 1995–1996 additions to the isoline collection of the USDA soybean genetic collection. Soybean Genetics Newsletter, 23, 43–50.

[tpg270056-bib-0005] Bolger, A. M. , Lohse, M. , & Usadel, B. (2014). Trimmomatic: A flexible trimmer for Illumina sequence data. Bioinformatics, 30, 2114–2120. 10.1093/bioinformatics/btu170 24695404 PMC4103590

[tpg270056-bib-0006] Brink, K. , Chui, C. F. , Cressman, R. F. , Garcia, P. , Henderson, N. , Hong, B. , Maxwell, C. A. , Meyer, K. , Mickelson, J. , Stecca, K. L. , Tyree, C. W. , Weber, N. , Zeng, W. , & Zhong, C. X. (2014). Molecular characterization, compositional analysis, and germination evaluation of a high‐oleic soybean generated by the suppression of expression. Crop Science, 54, 2160–2174. 10.2135/cropsci2012.06.0377

[tpg270056-bib-0007] Buhr, T. , Sato, S. , Ebrahim, F. , Xing, A. , Zhou, Y. , Mathiesen, M. , Schweiger, B. , Kinney, A. , Staswick, P. , & Clemente, T. (2002). Ribozyme termination of RNA transcripts down‐regulate seed fatty acid genes in transgenic soybean. Plant Journal, 30, 155–163. 10.1046/j.1365-313X.2002.01283.x 12000452

[tpg270056-bib-0008] Čermák, T. , Curtin, S. J. , Gil‐Humanes, J. , Čegan, R. , Kono, T. J. Y. , Konečná, E. , Belanto, J. J. , Starker, C. G. , Mathre, J. W. , Greenstein, R. L. , & Voytas, D. F. (2017). A multipurpose toolkit to enable advanced genome engineering in plants. Plant Cell, 29, 1196–1217. 10.1105/tpc.16.00922 28522548 PMC5502448

[tpg270056-bib-0009] Chiera, J. M. , Bouchard, R. A. , Dorsey, S. L. , Park, E. H. , Buenrostro‐Nava, M. T. , Ling, P. P. , & Finer, J. J. (2007). Isolation of two highly active soybean (*Glycine max* (L.) Merr.) promoters and their characterization using a new automated image collection and analysis system. Plant Cell Reporter, 26, 1501–1509. 10.1007/s00299-007-0359-y 17503049

[tpg270056-bib-0010] Demorest, Z. L. , Coffman, A. , Baltes, N. J. , Stoddard, T. J. , Clasen, B. M. , Luo, S. , Retterath, A. , Yabandith, A. , Gamo, M. E. , Bissen, J. , Mathis, L. , Voytas, D. F. , & Zhang, F. (2016). Direct stacking of sequence‐specific nuclease‐induced mutations to produce high oleic and low linolenic soybean oil. BMC Plant Biology, 16, 225. 10.1186/s12870-016-0906-1 27733139 PMC5062912

[tpg270056-bib-0011] Espina, M. J. C. , Lovell, J. T. , Jenkins, J. , Shu, S. , Sreedasyam, A. , Jordan, B. D. , Webber, J. , Boston, L. , Brůna, T. , Talag, J. , Goodstein, D. , Grimwood, J. , Stacey, G. , Cannon, S. B. , Lorenz, A. J. , Schmutz, J. , & Stupar, R. M. (2024). Assembly, comparative analysis, and utilization of a single haplotype reference genome for soybean. Plant Journal, 120, 1221–1235. 10.1111/tpj.17026 39276372

[tpg270056-bib-0012] Fauser, F. , Schiml, S. , & Puchta, H. (2014). Both CRISPR/Cas‐based nucleases and nickases can be used efficiently for genome engineering in *Arabidopsis thaliana* . Plant Journal, 79, 348–359. 10.1111/tpj.12554 24836556

[tpg270056-bib-0013] Gao, L. , Xie, L. , Xiao, Y. , Cheng, X. , Pu, R. , Zhang, Z. , Liu, Y. , Gao, S. , Zhang, Z. , Qu, H. , Zhi, H. , & Li, K. (2024). CRISPR/CasRx‐mediated resistance to soybean mosaic virus in soybean. Crop Journal, 12, 1093–1101. 10.1016/j.cj.2024.07.007

[tpg270056-bib-0014] Gillman, J. D. , Kim, W.‐S. , & Krishnan, H. B. (2015). Identification of a new soybean Kunitz trypsin inhibitor mutation and its effect on bowman‐birk protease inhibitor content in soybean seed. Journal of Agricultural and Food Chemistry, 63, 1352–1359. 10.1021/jf505220p 25608918

[tpg270056-bib-0015] Graham, N. , Patil, G. B. , Bubeck, D. M. , Dobert, R. C. , Glenn, K. C. , Gutsche, A. T. , Kumar, S. , Lindbo, J. A. , Maas, L. , May, G. D. , Vega‐Sanchez, M. E. , Stupar, R. M. , & Morrell, P. L. (2020). Plant genome editing and the relevance of off‐target changes. Plant Physiology, 183, 1453–1471. 10.1104/pp.19.01194 32457089 PMC7401131

[tpg270056-bib-0016] Gu, Z. , Gu, L. , Eils, R. , Schlesner, M. , & Brors, B. (2014). *circlize* Implements and enhances circular visualization in R. Bioinformatics, 30, 2811–2812. 10.1093/bioinformatics/btu393 24930139

[tpg270056-bib-0017] Haun, W. , Coffman, A. , Clasen, B. M. , Demorest, Z. L. , Lowy, A. , Ray, E. , Retterath, A. , Stoddard, T. , Juillerat, A. , Cedrone, F. , Mathis, L. , Voytas, D. F. , & Zhang, F. (2014). Improved soybean oil quality by targeted mutagenesis of the fatty acid desaturase 2 gene family. Plant Biotechnology Journal, 12, 934–940. 10.1111/pbi.12201 24851712

[tpg270056-bib-0018] Herman, E. M. , & Burks, A. W. (2011). The impact of plant biotechnology on food allergy. Current Opinion in Biotechnology, 22, 224–230. 10.1016/j.copbio.2010.11.003 21129947

[tpg270056-bib-0019] Joseph, L. M. , Hymowitz, T. , Schmidt, M. A. , & Herman, E. M. (2006). Evaluation of *Glycine* germplasm for nulls of the immunodominant allergen P34/Gly m Bd 30k. Crop Science, 46, 1755–1763. 10.2135/cropsci2005.12-0500

[tpg270056-bib-0020] Kim, S. , & Krishnan, H. B. (2023). A fast and cost‐effective procedure for reliable measurement of trypsin inhibitor activity in soy and soy products. Methods in Enzymology, 680, 195–213. 10.1016/bs.mie.2022.08.016 36710011

[tpg270056-bib-0021] Knowlton, S. (1999). Soybean oil having high oxidative stability . E.I. du Pont de Nemours and Company. U.S. Patent.

[tpg270056-bib-0022] Kshetry, A. O. , Ghose, K. , Alok, A. , Devkar, V. , Raman, V. , Stupar, R. M. , Herrera‐Estrella, L. , Zhang, F. , & Patil, G. B. (2025). Shoot at site: Advancing in planta transformation, regeneration and gene‐editing through a cascade of wounding‐mediated developmental regulators. bioRxiv. 10.1101/2025.02.06.636944

[tpg270056-bib-0023] Li, H. , & Durbin, R. (2009). Fast and accurate short read alignment with Burrows‐Wheeler transform. Bioinformatics, 25, 1754–1760. 10.1093/bioinformatics/btp324 19451168 PMC2705234

[tpg270056-bib-0024] Li, H. , Handsaker, B. , Wysoker, A. , Fennell, T. , Ruan, J. , Homer, N. , Marth, G. , Abecasis, G. , & Durbin, R. , & 1000 Genome Project Data Processing Subgroup . (2009). The sequence alignment/map format and SAMtools. Bioinformatics, 25, 2078–2079. 10.1093/bioinformatics/btp352 19505943 PMC2723002

[tpg270056-bib-0025] Liener, I. E. (1994). Implications of antinutritional components in soybean foods. Critical Reviews in Food Science and Nutrition, 34, 31–67. 10.1080/10408399409527649 8142044

[tpg270056-bib-0026] Liu, J. , Gunapati, S. , Mihelich, N. T. , Stec, A. O. , Michno, J.‐M. , & Stupar, R. M. (2019). Genome editing in soybean with CRISPR/Cas9. Methods in Molecular Biology, 1917, 217–234. 10.1007/978-1-4939-8991-1_16 30610639

[tpg270056-bib-0027] Liu, K. (2024). Enzymatic and algebraic methodology to determine the contents of Kunitz and Bowman‐Birk Inhibitors and their contributions to total trypsin or chymotrypsin inhibition in soybeans. Journal of Agricultural and Food Chemistry, 72, 11782–11793. 10.1021/acs.jafc.3c06389 38717295 PMC11122080

[tpg270056-bib-0028] Mooney, B. P. , Krishnan, H. B. , & Thelen, J. J. (2004). High‐throughput peptide mass fingerprinting of soybean seed proteins: Automated workflow and utility of UniGene expressed sequence tag databases for protein identification. Phytochemistry, 65, 1733–1744. 10.1016/j.phytochem.2004.04.011 15276434

[tpg270056-bib-0029] Mroczka, A. , Roberts, P. D. , Fillatti, J. J. , Wiggins, B. E. , Ulmasov, T. , & Voelker, T. (2010). An intron sense suppression construct targeting soybean FAD2‐1 requires a double‐stranded RNA‐producing inverted repeat T‐DNA insert. Plant Physiology, 153, 882–891. 10.1104/pp.110.154351 20424004 PMC2879809

[tpg270056-bib-0030] Oliva, R. , Ji, C. , Atienza‐Grande, G. , Huguet‐Tapia, J. C. , Perez‐Quintero, A. , Li, T. , Eom, J.‐S. , Li, C. , Nguyen, H. , Liu, B. , Auguy, F. , Sciallano, C. , Luu, V. T. , Dossa, G. S. , Cunnac, S. , Schmidt, S. M. , Slamet‐Loedin, I. H. , Vera Cruz, C. , Szurek, B. , … Yang, B. (2019). Broad‐spectrum resistance to bacterial blight in rice using genome editing. Nature Biotechnology, 37, 1344–1350. 10.1038/s41587-019-0267-z PMC6831514 31659337

[tpg270056-bib-0031] Paz, M. M. , Martinez, J. C. , Kalvig, A. B. , Fonger, T. M. , & Wang, K. (2006). Improved cotyledonary node method using an alternative explant derived from mature seed for efficient *Agrobacterium*‐mediated soybean transformation. Plant Cell Reports, 25, 206–213. 10.1007/s00299-005-0048-7 16249869

[tpg270056-bib-0032] Paz, M. M. , Shou, H. , Guo, Z. , Zhang, Z. , Banerjee, A. K. , & Wang, K. (2004). Assessment of conditions affecting *Agrobacterium*‐mediated soybean transformation using the cotyledonary node explant. Euphytica, 136, 167–179. 10.1023/B:EUPH.0000030670.36730.a4

[tpg270056-bib-0033] Pham, A. T. , Lee, J. D. , Shannon, J. G. , & Bilyeu, K. (2010). Mutant alleles of *fad2‐1a* and *fad2‐1b* combine to produce soybeans with the high oleic acid seed oil trait. BMC Plant Biology, 10, Article 195. 10.1186/1471-2229-10-195 20828382 PMC2956544

[tpg270056-bib-0034] Satterlee, J. W. , Alonso, D. , Gramazio, P. , Jenike, K. M. , He, J. , Arrones, A. , Villanueva, G. , Plazas, M. , Ramakrishnan, S. , Benoit, M. , Gentile, I. , Hendelman, A. , Shohat, H. , Fitzgerald, B. , Robitaille, G. M. , Green, Y. , Swartwood, K. , Passalacqua, M. J. , Gagnon, E. , … Lippman, Z. B. (2024). Convergent evolution of plant prickles by repeated gene co‐option over deep time. Science, 385, eado1663. 10.1126/science.ado1663 39088611 PMC11305333

[tpg270056-bib-0035] Saurabh, S. , Vidyarthi, A. S. , & Prasad, D. (2014). RNA interference: Concept to reality in crop improvement. Planta, 239, 543–564. 10.1007/s00425-013-2019-5 24402564

[tpg270056-bib-0036] Schmidt, M. A. , Hymowitz, T. , & Herman, E. M. (2015). Breeding and characterization of soybean *Triple Null*; a stack of recessive alleles of Kunitz trypsin inhibitor, soybean agglutinin, and P34 allergen nulls. Plant Breeding, 134, 310–315. 10.1111/pbr.12265

[tpg270056-bib-0037] Schmutz, J. , Cannon, S. B. , Schlueter, J. , Ma, J. , Mitros, T. , Nelson, W. , Hyten, D. L. , Song, Q. , Thelen, J. J. , Cheng, J. , Xu, D. , Hellsten, U. , May, G. D. , Yu, Y. , Sakurai, T. , Umezawa, T. , Bhattacharyya, M. K. , Sandhu, D. , Valliyodan, B. , … Jackson, S. A. (2010). Genome sequence of the palaeopolyploid soybean. Nature, 463, 178–183. 10.1038/nature08670 20075913

[tpg270056-bib-0038] Shaheen, N. , Ahmad, S. , Alghamdi, S. S. , Rehman, H. M. , Javed, M. A. , Tabassum, J. , & Shao, G. (2023). CRISPR‐Cas system, a possible “savior” of rice threatened by climate change: An updated review. Rice, 16, 39. 10.1186/s12284-023-00652-1 37688677 PMC10492775

[tpg270056-bib-0039] Song, Q. , Jenkins, J. , Jia, G. , Hyten, D. L. , Pantalone, V. , Jackson, S. A. , Schmutz, J. , & Cregan, P. B. (2016). Construction of high resolution genetic linkage maps to improve the soybean genome sequence assembly Glyma1.01. BMC Genomics, 17, Article 33. 10.1186/s12864-015-2344-0 26739042 PMC4704267

[tpg270056-bib-0040] Srivastava, A. , Philip, V. M. , Greenstein, I. , Rowe, L. B. , Barter, M. , Lutz, C. , & Reinholdt, L. G. (2014). Discovery of transgene insertion sites by high throughput sequencing of mate pair libraries. BMC Genomics, 15, Article 367. 10.1186/1471-2164-15-367 24884803 PMC4035081

[tpg270056-bib-0041] Sweeney, D. W. , Carrero‐Colón, M. , & Hudson, K. A. (2017). Characterization of new allelic combinations for high‐oleic soybeans. Crop Science, 57, 611–616. 10.2135/cropsci2015.09.0596

[tpg270056-bib-0042] Valliyodan, B. , Cannon, S. B. , Bayer, P. E. , Shu, S. , Brown, A. V. , Ren, L. , Jenkins, J. , Chung, C. Y.‐L. , Chan, T.‐F. , Daum, C. G. , Plott, C. , Hastie, A. , Baruch, K. , Barry, K. W. , Huang, W. , Patil, G. , Varshney, R. K. , Hu, H. , Batley, J. , … Nguyen, H. T. (2019). Construction and comparison of three reference‐quality genome assemblies for soybean. Plant Journal, 100, 1066–1082. 10.1111/tpj.14500 31433882

[tpg270056-bib-0043] van der Auwera, G. , & O'Connor, B. D. (2020). Genomics in the Cloud: Using Docker, GATK, and WDL in Terra. O'Reilly Media.

[tpg270056-bib-0044] Virdi, K. S. , Spencer, M. , Stec, A. O. , Xiong, Y. , Merry, R. , Muehlbauer, G. J. , & Stupar, R. M. (2020). Similar seed composition phenotypes are observed from CRISPR‐generated in‐frame and knockout alleles of a soybean KASI ortholog. Frontiers in Plant Science, 11, 1005. 10.3389/fpls.2020.01005 32774339 PMC7381328

[tpg270056-bib-0045] Wang, Z. , Shea, Z. , Rosso, L. , Shang, C. , Li, J. , Bewick, P. , Li, Q. , Zhao, B. , & Zhang, B. (2023). Development of new mutant alleles and markers for KTI1 and KTI3 via CRISPR/Cas9‐mediated mutagenesis to reduce trypsin inhibitor content and activity in soybean seeds. Frontiers in Plant Science, 14, 1111680. 10.3389/fpls.2023.1111680 37223818 PMC10200896

[tpg270056-bib-0046] Zhang, J. , Lyu, H. , Chen, J. , Cao, X. , Du, R. , Ma, L. , Wang, N. , Zhu, Z. , Rao, J. , Wang, J. , Zhong, K. , Lyu, Y. , Wang, Y. , Lin, T. , Zhou, Y. , Zhou, Y. , Zhu, G. , Fei, Z. , Klee, H. , & Huang, S. (2024). Releasing a sugar brake generates sweeter tomato without yield penalty. Nature, 635, 647–656. 10.1038/s41586-024-08186-2 39537922 PMC11578880

[tpg270056-bib-0047] Zhang, X. , Smits, A. H. , van Tilburg, G. B. , Ovaa, H. , Huber, W. , & Vermeulen, M. (2018). Proteome‐wide identification of ubiquitin interactions using UbIA‐MS. Nature Protocols, 13, 530–550. 10.1038/nprot.2017.147 29446774

[tpg270056-bib-0048] Zhang, Y. , Bai, Y. , Wu, G. , Zou, S. , Chen, Y. , Gao, C. , & Tang, D. (2017). Simultaneous modification of three homoeologs of TaEDR1 by genome editing enhances powdery mildew resistance in wheat. Plant Journal, 91, 714–724. 10.1111/tpj.13599 28502081

[tpg270056-bib-0049] Zhong, H. , Li, C. , Yu, W. , Zhou, H. P. , Lieber, T. , Su, X. , Wang, W. , Bumann, E. , Lunny Castro, R. M. , Jiang, Y. , Gu, W. , Liu, Q. , Barco, B. , Zhang, C. , Shi, L. , & Que, Q. (2024). A fast and genotype‐independent in planta Agrobacterium‐mediated transformation method for soybean. Plant Communications, 5, 101063. 10.1016/j.xplc.2024.101063 39138866 PMC11671754

[tpg270056-bib-0050] Zhu, X. , Xu, Y. , Yu, S. , Lu, L. , Ding, M. , Cheng, J. , Song, G. , Gao, X. , Yao, L. , Fan, D. , Meng, S. , Zhang, X. , Hu, S. , & Tian, Y. (2014). An efficient genotyping method for genome‐modified animals and human cells generated with CRISPR/Cas9 system. Scientific Reports, 4, 6420. 10.1038/srep06420 25236476 PMC4168274

