# Supplementary material for: Genomic and biochemical comparison of allelic triple‐mutant lines derived from conventional breeding and multiplex gene editing
Source: Plant Genome. 2025 Jun 5;18(2):e70056. doi: 10.1002/tpg2.70056 (PMC12141651; doi:10.1002/tpg2.70056)
Supplement: Supplementary file 1 [file TPG2-18-e70056-s003.pdf]

(A)

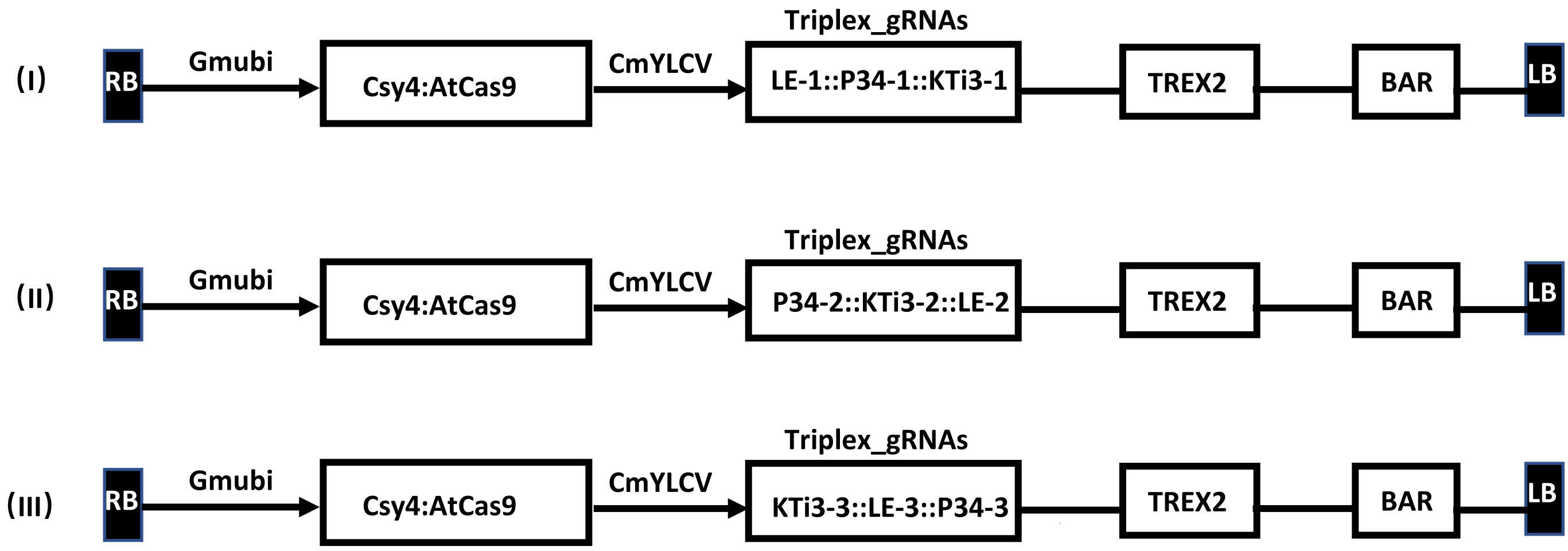

(B)

(I)	**LE-1**	**P34-1**	**KTi3-1**
	GATCCATCAAAACGACGTCTTGG	GGCCAACAAGAAAATGAAGAAGG	GTTGTGGAGGATCTACCAGAAGG
(II)	**P34-2**	**KTi3-2**	**LE-2**
	GCAGCACATGCAATAGCAACAGG	GAATCGAACTTAAGGCTCAAAGG	GAAGACTTCTCTTCCCGAGTGGG
(III)	**KTi3-3**	**LE-3**	**P34-3**
	GATGGCCTTCGGCGATAAAACGG	GTTTGTGGCTTAGTGTCAATTGG	GGCACTATCAATCGTTCGAATGG

Figure S1. Schematic representation of T-DNA regions of the CRISPR constructs used in this study. (A) Diagram of the transgene components in three constructs. They all include a Cas9 gene fused to Csy4, a TREX2 enzyme, a BAR selectable marker, and three gRNA sequences targeting the respective three genes (labeled as LE, P34, and KTi3 here). Note that the position of the three gRNAs is shifted among the three constructs. (B) The gRNA sequences used in each of the three constructs.

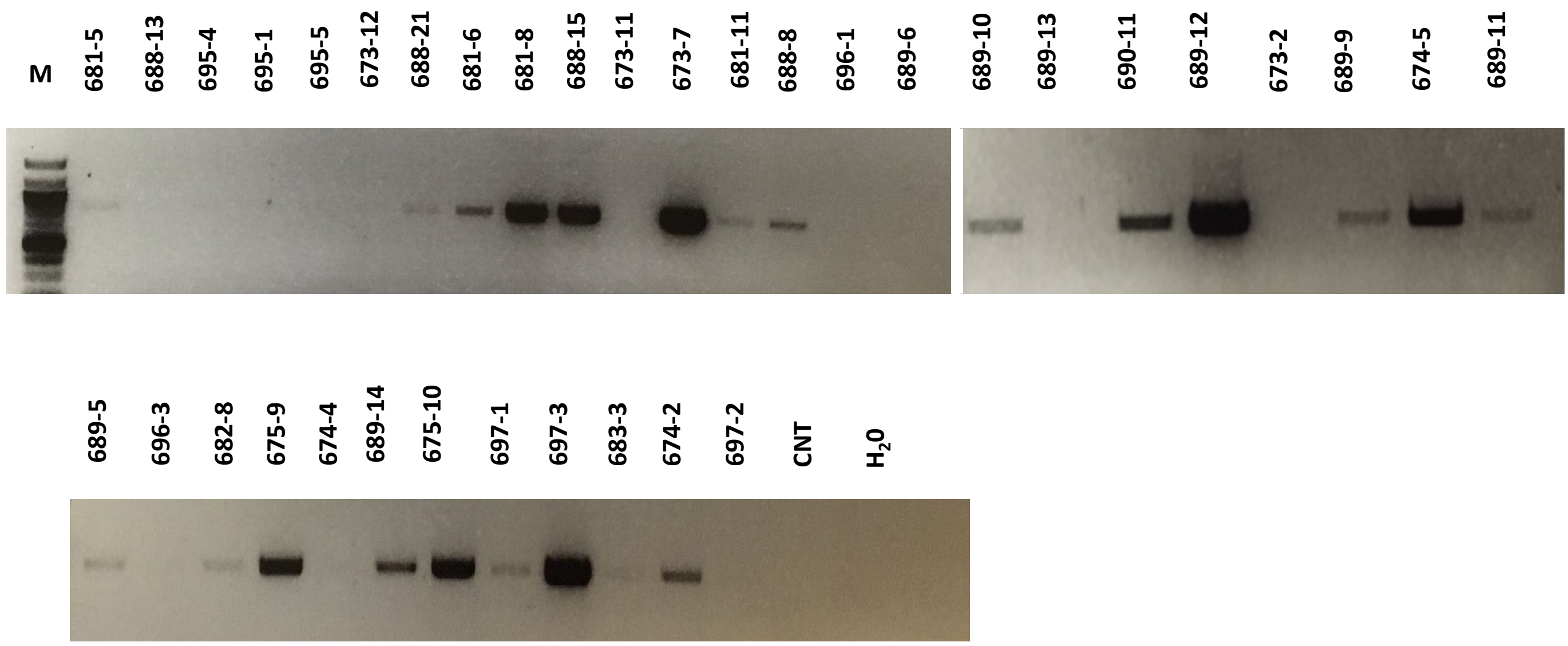

Figure S2: PCR amplification of transgenes from regenerated soybean plants. Genomic DNA was extracted from young leaves of regenerated putative T_0 soybean plants. PCR amplification of transgenes was performed with GmUbi/Cas9 primers spanning the junction between GmUbi promoter and AtCas9 coding region. CNT: Control non-transgenic soybean plant. H_2O: water as a negative PCR control. The vertical white bar in the upper image indicates where images were spliced together to simplify the visual comparison of samples.

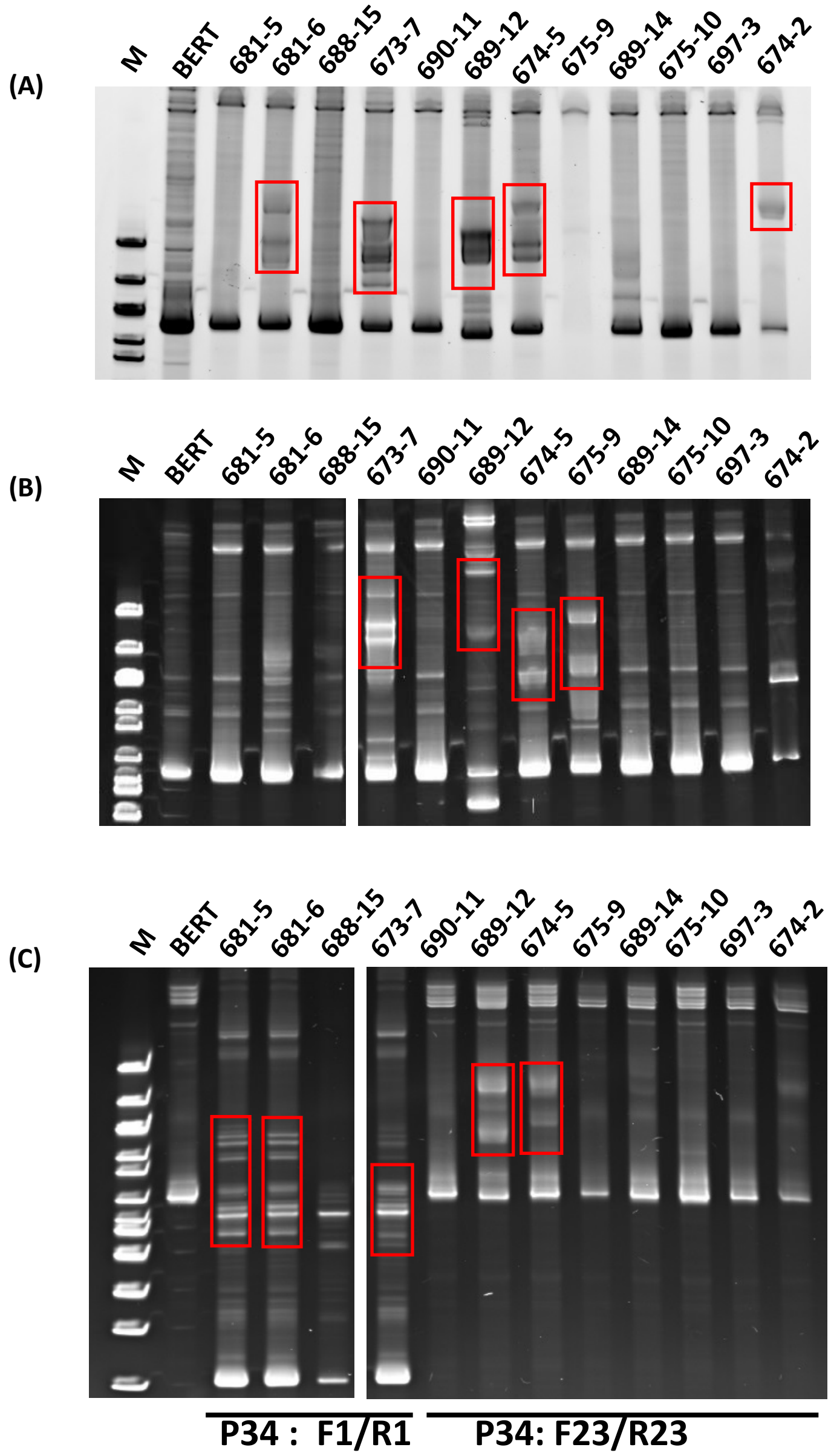

Figure S3. Preliminary screening of somatic mutations in the primary transformants (T_0). PCR amplified target regions from the LE, KTi3, and P34 genes were resolved in gradient 4-20% (1 x TBE) polyacrylamide gels. PCR amplicons with heteroduplex DNA of three target genes were displayed for LE (A), KTi3 (B), and P34 (C), respectively. The WPT pre-fix was left off the lane labels for simplicity of viewing. The vertical white bars dividing lanes 688-15 and 673-7 in parts (B) and (C) indicate where images were spliced together to simplify the visual comparison of samples. Red rectangles indicate the presence of mutations, as altered mobility is presumably caused by heteroduplex formation in the annealed PCR product. The T_0 plants (WPT673-7, WPT689-12, and WPT674-5) appeared to contain mutations in all three target genes. M: Molecular weight size marker.

(A) WPT673-7

Gene	Allele	Sequence
LE	WT	CACATCGGAATTAACGTCAATTCTATCAGATCCATCAAAACGACGTCTTGGGATTTGGCCAACAATAAAGTAGC
	Δ4	CACATCGGAATTAACGTCAATTCTATCAGATCCATCAAAAC----TCTTGGGATTTGGCCAACAATAAAGTAGC
	Δ11	CACATCGGAATTAACGTCAATTCTATCAGATCCATC-----------TTGGGATTTGGCCAACAATAAAGTAGC
KTi3	WT	ATGCTGTGTGTTGGAATTCCTACCGAGTGGTCTGTTGTGGAGGATCTACCAGAAGGACCTGCTGTTAAAATT
	Δ5	ATGCTGTGTGTTGGAATTCCTACCGAGTGGTCTGTTGTGGAGGAT-----AGAAGGACCTGCTGTTAAAATT
	Δ12	ATGCTGTGTGTTGGAATTCCTACCGAGTGGTCTGTTGTGGAGG------------GACCTGCTGTTAAAATT
P34	WT	TCGCAGCAAATCAAAATGGCCAACAAGAAAATGAAGAAGGAACAATATTCTTGTGACCATCCACCTGCA
	Δ3	TCGCAGCAAATCAAAATGGCCAACAAGAAAA---AGAAGGAACAATATTCTTGTGACCATCCACCTGCA
	Δ7	TCGCAGCAAATCAAAATGGCCAACAAGAA-------AAGGAACAATATTCTTGTGACCATCCACCTGCA

(B) WPT673-10

Gene	Allele	Sequence
LE	WT	CACATCGGAATTAACGTCAATTCTATCAGATCCATCAAAACGACGTCTTGGGATTTGGCCAACAATAAAGTAGC
	Δ16	CACATCGGAATTAACGTCAATTCTATCAGA----------------CTTGGGATTTGGCCAACAATAAAGTAGC
	Δ17	CACATCGGAATTAACGTCAATTCTATCAGA-----------------TTGGGATTTGGCCAACAATAAAGTAGC
KTi3	WT	ATGCTGTGTGTTGGAATTCCTACCGAGTGGTCTGTTGTGGAGGATCTACCAGAAGGACCTGCTGTTAAAATT
	Δ2	ATGCTGTGTGTTGGAATTCCTACCGAGTGGTCTGTTGTGGAGGATCTA--AGAAGGACCTGCTGTTAAAATT
	Δ15	ATGCTGTGTGTTGGAATTCCTACCGAGTGGTCTGT---------------AGAAGGACCTGCTGTTAAAATT
P34	WT	TCGCAGCAAATCAAAATGGCCAACAAGAAAATGAAGAAGGAACAATATTCTTGTGACCATCCACCTGCA
	Δ4	TCGCAGCAAATCAAAATGGCCAACAAGA----GAAGAAGGAACAATATTCTTGTGACCATCCACCTGCA
	Δ4	TCGCAGCAAATCAAAATGGCCAACAAGA----GAAGAAGGAACAATATTCTTGTGACCATCCACCTGCA

(C) WPT689-12

Gene	Allele	Sequence
LE	WT	CAATATCCTCTCCGATGTGGTCGATTTGAAGACTTCTCTTCCCGAGTGGGTGAGGATAGGGTTCTCTGCT
	Δ14	CAATATCCTCTCCGATGTGGTCGATTTGAA--------------AGTGGGTGAGGATAGGGTTCTCTGCT
	Δ32	CAATATCCTCTCCGATGTGGTC--------------------------------GATAGGGTTCTCTGCT
KTi3	WT	TCCAACACACAGCATTATAACTGCAAATGAATCGAACTTAAGGCTCAAAGGATGGCCTTCGGCGAT
	Δ7	TCCAACACACAGCATTATAACTGCAAATGAATCGAACT-------CAAAGGATGGCCTTCGGCGAT
	Δ104	TCCAACACACAGCATTAT-----------------------------------//--TGCGAGATTG
P34	WT	TTCTGCCACGGGAGCCATAGAAGCAGCACATGCAATAGCAACAGGAGACCTTGTTAGCCTTT
	Δ3	TTCTGCCACGGGAGCCATAGAAGCAGCACATGCAAT---AACAGGAGACCTTGTTAGCCTTT
	Δ12	TTCTGCCACGGGAGCCATAGAAGCAGC------------AACAGGAGACCTTGTTAGCCTTT

Figure S4. Heritable mutations transmitted from the T_0 to the T_1 generation. Note that WPT689-12 exhibited only in-frame mutations (3 bp and 12 bp deletions) in the P34 target. Meanwhile, the WPT673-7 progeny showed evidence for inheritance of frameshift alleles for all three target genes.

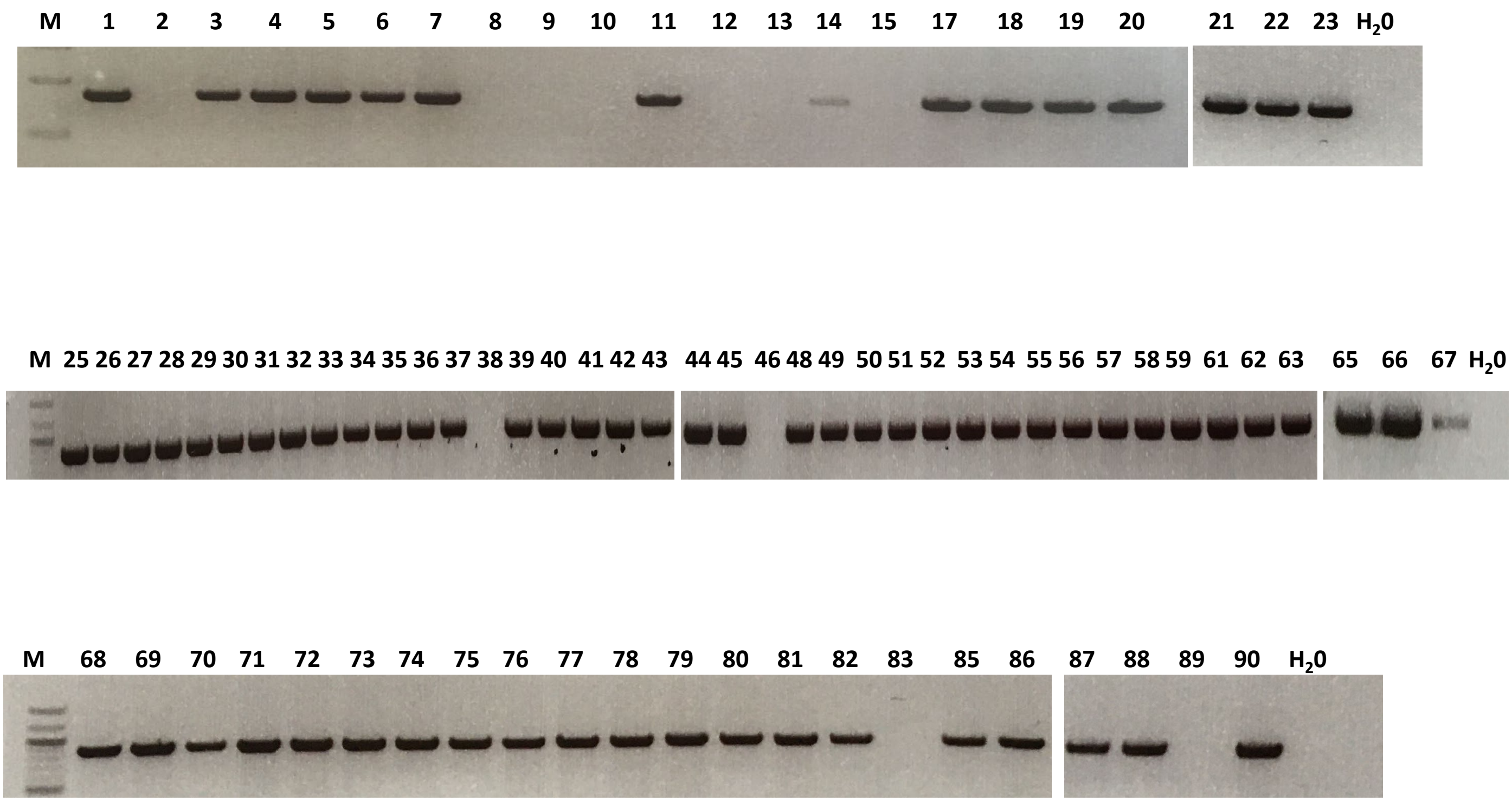

Figure S5. Segregation of transgenes in T_1 progenies of event WPT673-7. Genomic DNA was extracted from young leaves of T_1 plants derived from WPT673-7. PCR amplification of transgenes was performed with GmUbi/Cas9 primers spanning the junction between GmUbi promoter and AtCas9 coding region. Sample numbers were serial numbers of T_1 plants preceded with WPT673-7-. Ten out of ninety T_1 plants were transgene-negative: WPT 673-7-2, 673-7-8, 673-7-9, 673-7-12, 673-7-13, 673-7-15, 673-7-38, 673-7-46, 673-7-83 and 673-7-89; H_2O: water as negative PCR control; M: Molecular weight size marker. The vertical white bars indicate where gel images were spliced together to simplify the comparison of samples.

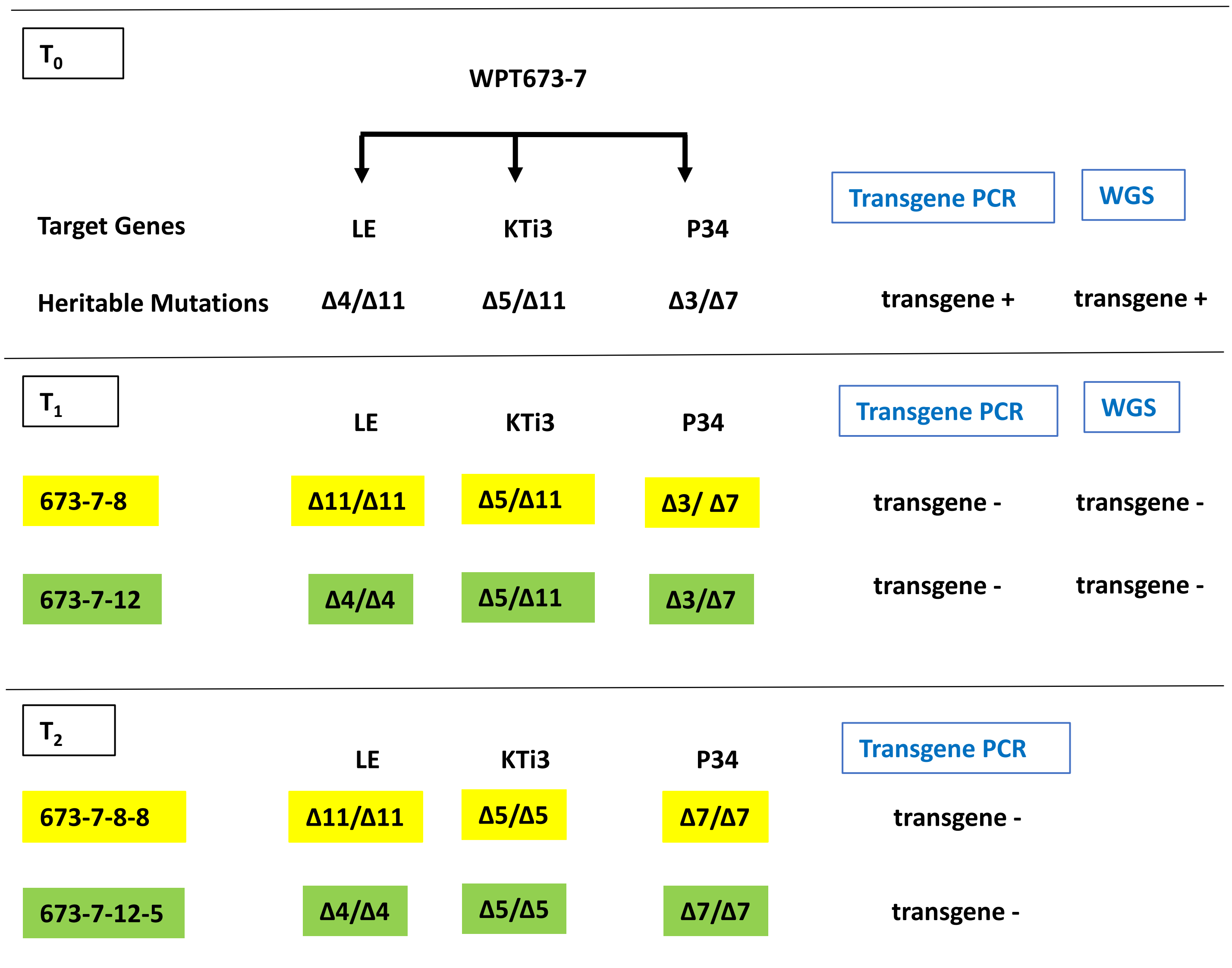

Figure S6. Selection of T_2 progenies harboring homozygous mutations with potential knock-out effects for all three target genes. The presence of transgene sequences were screened using PCR and whole-genome sequencing in the T_0 and T_1 plants. The non-transgenic status of the two T_2 plants was confirmed with PCR and whole-genome sequencing.

(A)

WPT673-7-8-8

LE

```
WT   CACATCGGAATTAACGTCAATTCTATCAGATCCATCAAAACGACGTCTTGGGATTTGGCCAACAATAAAGTAGC
Δ11  CACATCGGAATTAACGTCAATTCTATCAGATCCATC-----------TTGGGATTTGGCCAACAATAAAGTAGC
Δ11  CACATCGGAATTAACGTCAATTCTATCAGATCCATC-----------TTGGGATTTGGCCAACAATAAAGTAGC
```

KTi3

```
WT   ATGCTGTGTGTTGGAATTCCTACCGAGTGGTCTGTTGTGGAGGATCTACCAGAAGGACCTGCTGTTAAAATT
Δ5   ATGCTGTGTGTTGGAATTCCTACCGAGTGGTCTGTTGTGGAGGAT-----AGAAGGACCTGCTGTTAAAATT
Δ5   ATGCTGTGTGTTGGAATTCCTACCGAGTGGTCTGTTGTGGAGGAT-----AGAAGGACCTGCTGTTAAAATT
```

P34

```
WT   TCGCAGCAAATCAAAATGGCCAACAAGAAAATGAAGAAGGAACAATATTCTTGTGACCATCCACCTGCA
Δ7   TCGCAGCAAATCAAAATGGCCAACAAGAA-------AAGGAACAATATTCTTGTGACCATCCACCTGCA
Δ7   TCGCAGCAAATCAAAATGGCCAACAAGAA-------AAGGAACAATATTCTTGTGACCATCCACCTGCA
```

(B)

WPT673-7-12-5

LE

```
WT   CACATCGGAATTAACGTCAATTCTATCAGATCCATCAAAACGACGTCTTGGGATTTGGCCAACAATAAAGTAGC
Δ4   CACATCGGAATTAACGTCAATTCTATCAGATCCATCAAAAC----TCTTGGGATTTGGCCAACAATAAAGTAGC
Δ4   CACATCGGAATTAACGTCAATTCTATCAGATCCATCAAAAC----TCTTGGGATTTGGCCAACAATAAAGTAGC
```

KTi3

```
WT   ATGCTGTGTGTTGGAATTCCTACCGAGTGGTCTGTTGTGGAGGATCTACCAGAAGGACCTGCTGTTAAAATT
Δ5   ATGCTGTGTGTTGGAATTCCTACCGAGTGGTCTGTTGTGGAGGAT-----AGAAGGACCTGCTGTTAAAATT
Δ5   ATGCTGTGTGTTGGAATTCCTACCGAGTGGTCTGTTGTGGAGGAT-----AGAAGGACCTGCTGTTAAAATT
```

P34

```
WT   TCGCAGCAAATCAAAATGGCCAACAAGAAAATGAAGAAGGAACAATATTCTTGTGACCATCCACCTGCA
Δ7   TCGCAGCAAATCAAAATGGCCAACAAGAA-------AAGGAACAATATTCTTGTGACCATCCACCTGCA
Δ7   TCGCAGCAAATCAAAATGGCCAACAAGAA-------AAGGAACAATATTCTTGTGACCATCCACCTGCA
```

Figure S7. Mutation profiles of LE, KTi3 and P34 genes in the T_2 lines used for protein/proteomic analysis in this study. Both of these plants were non-transgenic and carried homozygous frameshift alleles for all three target genes.

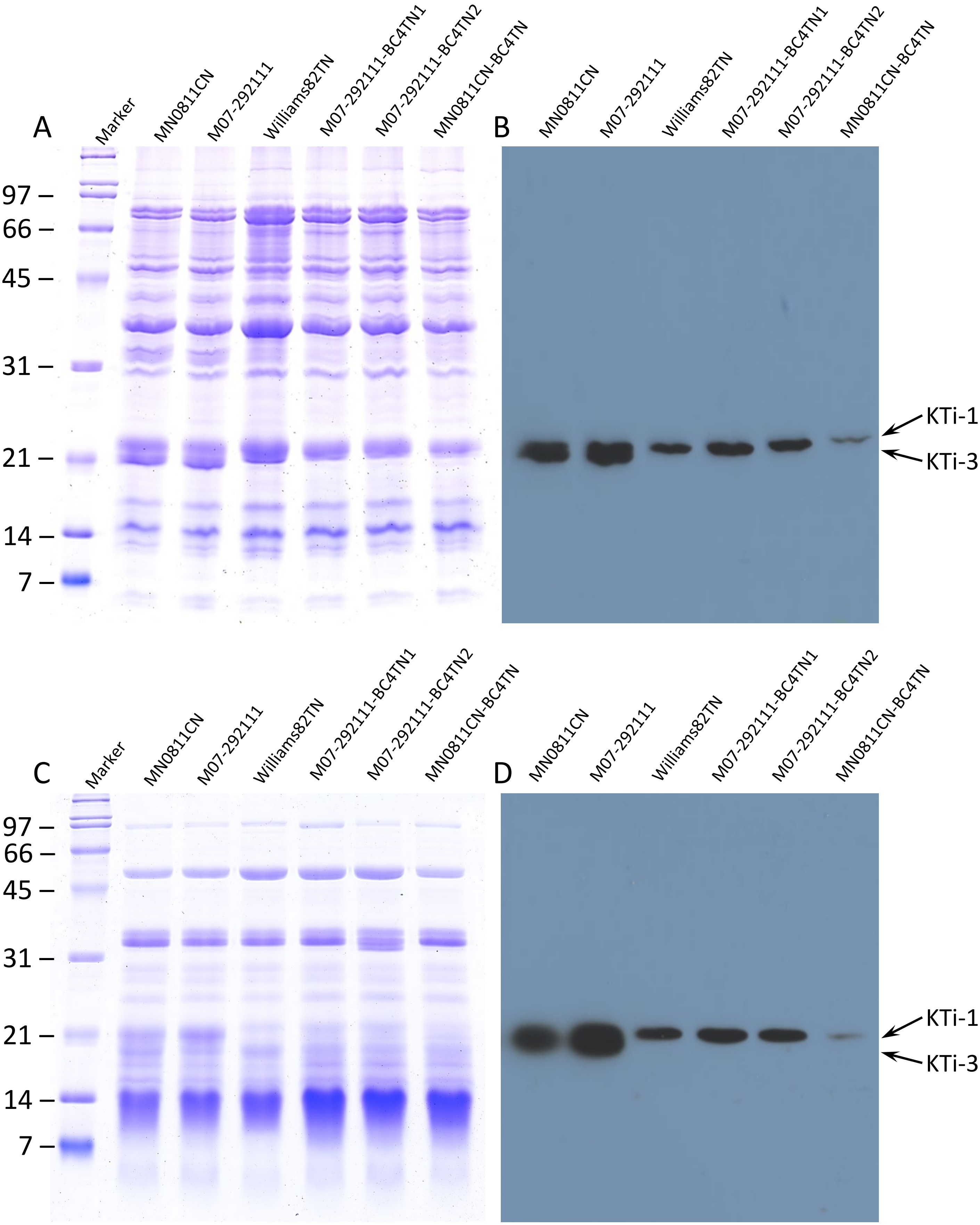

Figure S8. Detection of KTi-1 and KTi-3 proteins in soybean seeds for conventionally-bred triple-mutants compared to recurrent parents. (A-B) Seed proteins remaining in the supernatant after fractionation with 100 mM calcium chloride were resolved by 13.5% SDS-PAGE. (C-D) Isopropanol extracted seed proteins were resolved by 13.5% SDS-PAGE. Panel A, C: Coomassie Blue stained gel. Panel B, D: Immunoblot analysis with soybean KTi antibodies. Marker = Protein Molecular weight markers.

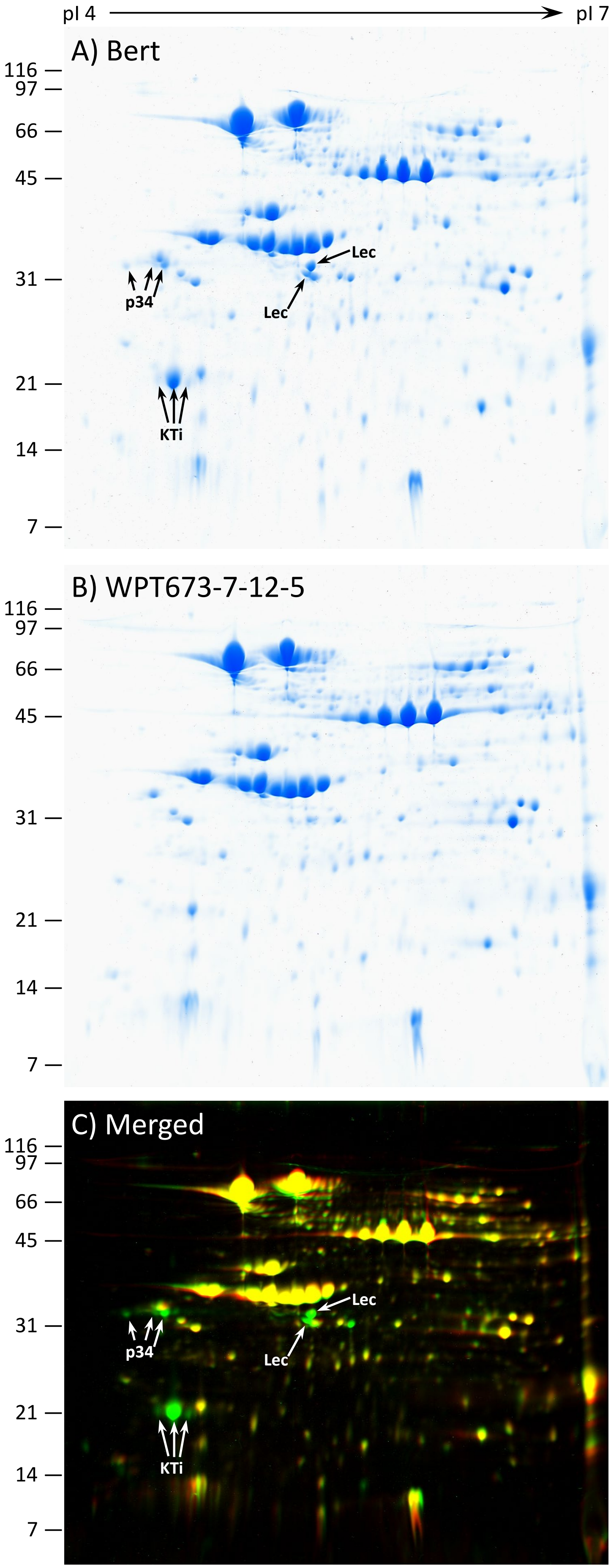

Figure S9. Two-dimensional gel electrophoresis of soybean seed proteins in WPT673-7-12-5 compared to Bert. Seed proteins (300 µg) were separated by isoelectric focusing on pI 4-7 strips, followed by SDS-PAGE on 10-16% gradient gels. Following electrophoresis, the gels were stained with Colloidal Coomassie Blue G-250. The position and sizes of the protein molecular weight markers in kDa are shown on the left side of the figure. (A) Bert; (B) WPT673-7-12-5. (C) Overlay of two separate two-dimensional gels of soybean seed proteins using Delta2D software. Gels were scanned and resulting images were assigned two different colors (green = Bert; red = WPT673-7-12-5) to visualize the differences between the two. Yellow demonstrates similar protein quantities in each. Green color demonstrates absence of that protein species in the WPT673-7-12-5 mutant. Arrows point to proteins missing in WPT673-7-12-5.

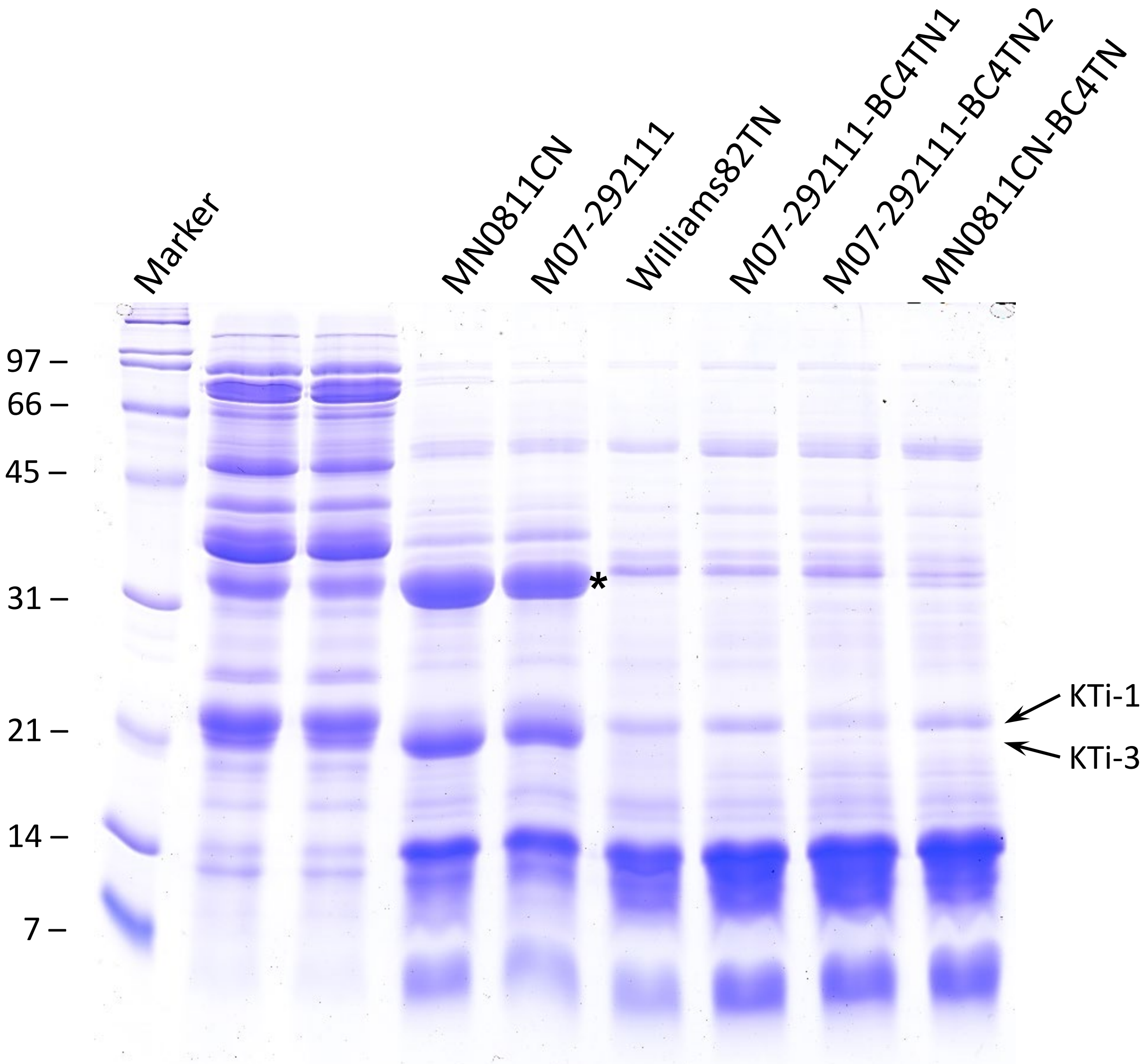

Figure S10: SDS-PAGE analysis of ethanol-extracted soybean seed proteins for conventionally-bred triple-mutants compared to recurrent parents. Proteins were separated with 15% SDS-PAGE and visualized by staining the gel with Coomassie Blue. M = Protein Molecular weight markers. The * symbol indicates the location of P34 and lectin proteins.

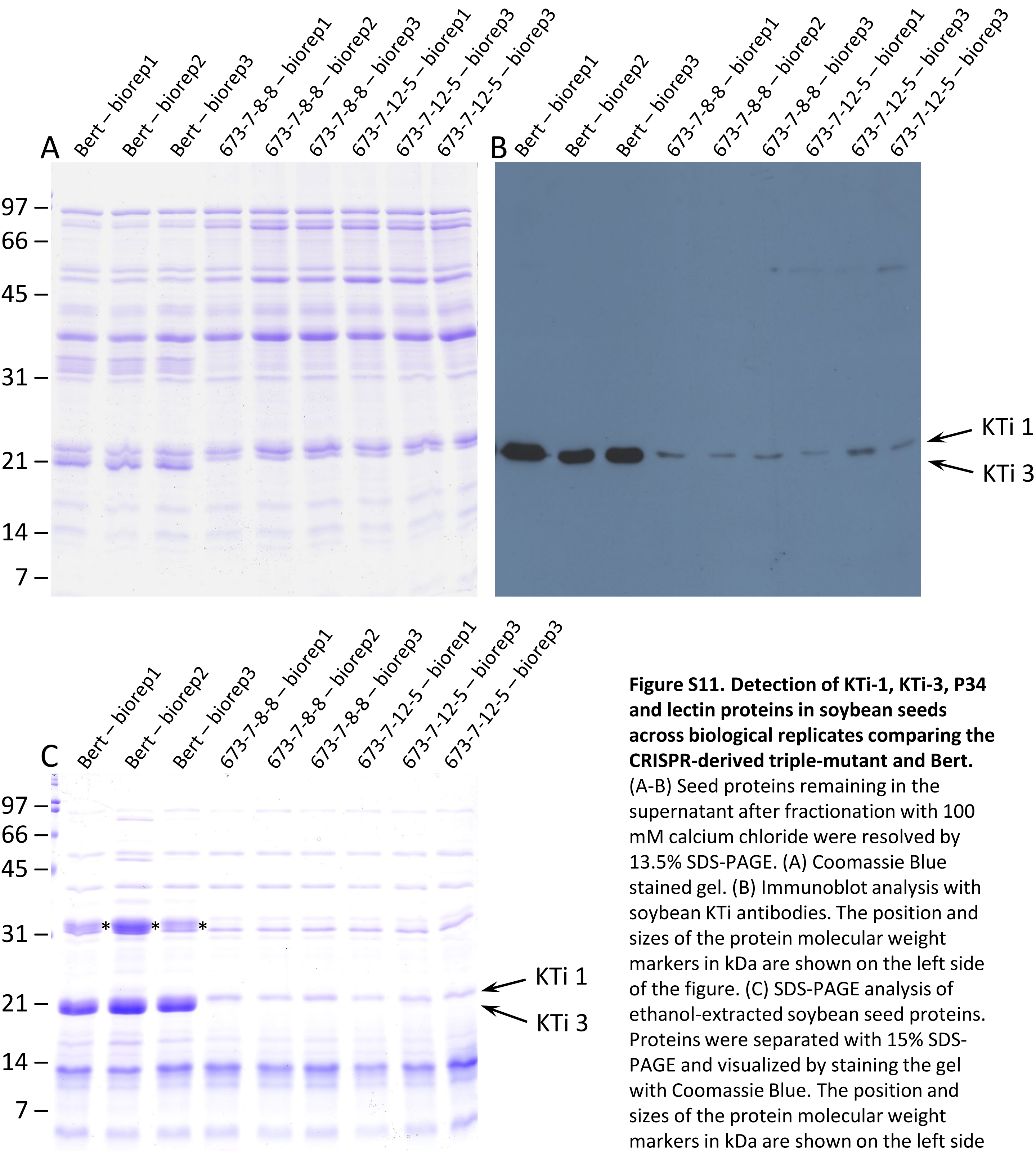

Figure S11. Detection of KTi-1, KTi-3, P34 and lectin proteins in soybean seeds across biological replicates comparing the CRISPR-derived triple-mutant and Bert. (A-B) Seed proteins remaining in the supernatant after fractionation with 100 mM calcium chloride were resolved by 13.5% SDS-PAGE. (A) Coomassie Blue stained gel. (B) Immunoblot analysis with soybean KTi antibodies. The position and sizes of the protein molecular weight markers in kDa are shown on the left side of the figure. (C) SDS-PAGE analysis of ethanol-extracted soybean seed proteins. Proteins were separated with 15% SDS-PAGE and visualized by staining the gel with Coomassie Blue. The position and sizes of the protein molecular weight markers in kDa are shown on the left side of the figure. The * symbol indicates the location of P34 and lectin proteins.

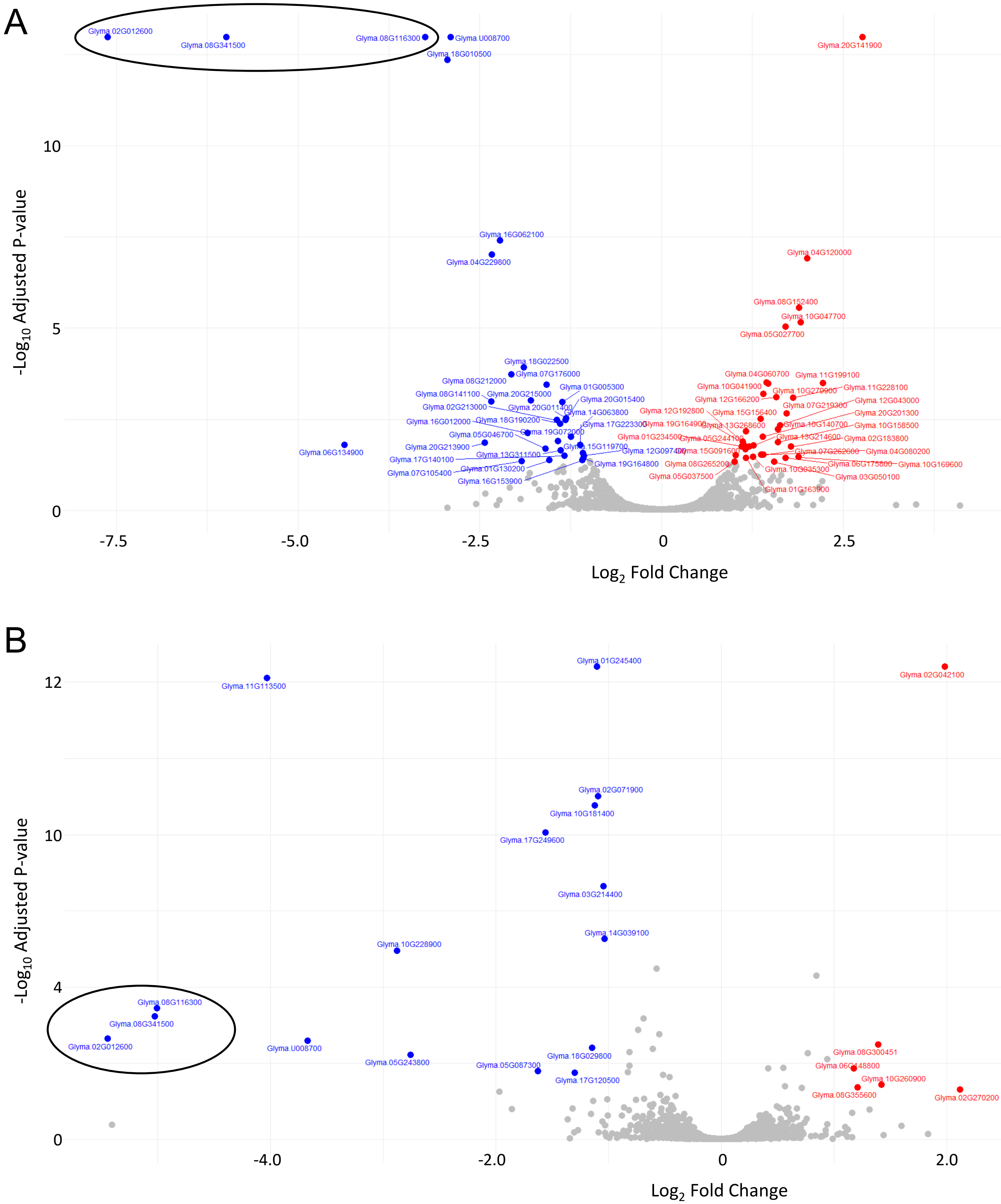

Figure S12. Differentially expressed proteins (DEPs) in R8 soybean seeds. (A) DEPs in conventionally-bred triple-mutant line MN0811CN-BC4TN compared to the recurrent parent MN0811CN. (B) DEPs in CRISPR-derived triple-mutants (shown as the average of WPT673-7-12-5 and WPT673-7-8-8) compared to Bert. Blue dots represent downregulated proteins in the triple-mutants while red dots represent upregulated proteins. The three triple-mutant gene models are circled in both images, indicating their strong downregulation. Fold change is shown as log_2 values.

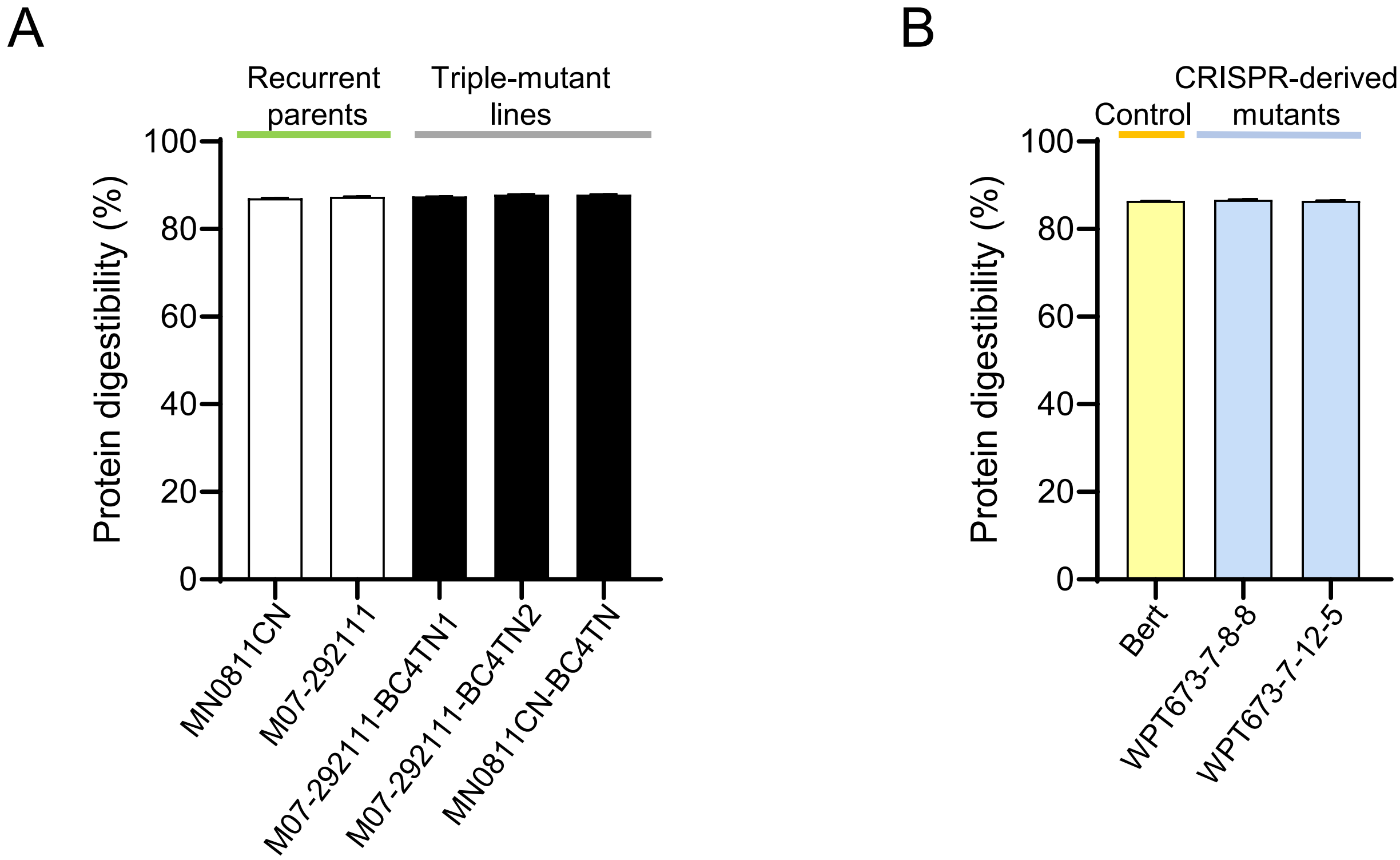

Figure S13. Protein digestibility was not affected in triple-mutants. Protein digestibility in conventionally-bred triple-mutant lines (A) and CRISPR-derived triple-mutant lines (B). MN0811CN, M07-292111 (recurrent parent controls), M07-292111-BC4TN1, M07-292111-BC4TN2, and MN0811CN-BC4TN (three conventionally-bred triple-mutants), Bert (Control), WPT673-7-8-8 and WPT673-7-12-5 (two CRISPR-derived triple-mutants in the Bert background). Each genotype value represents the average of three technical replicates of ~100 bulk seeds from 20-25 field-harvested plants per genotype. Error bars indicate SD. One-way ANOVA analysis showed no significant difference among different genotypes.
