# Supplementary material for: Genomic and biochemical comparison of allelic triple‐mutant lines derived from conventional breeding and multiplex gene editing
Source: Plant Genome. 2025 Jun 5;18(2):e70056. doi: 10.1002/tpg2.70056 (PMC12141651; doi:10.1002/tpg2.70056)
Supplement: Supplementary file 2 [file TPG2-18-e70056-s002.docx]

**Table S1.** Oligos for cloning 3plex gRNAs

**3plex-I**

Csy4-B_gRNA1-1: TCG TCT CCT TTT GAT GGA TCC TGC CTA TAC GGC AGT GAA CCT G
Rep-C_gRNA1-1: TCG TCT CAA AAA CGA CGT CTG TTT TAG AGC TAG AAA TAG C
Csy-D_gRNA1-2: TCG TCT CAT TCT TGT TGG CCC TGC CTA TAC GGC AGT GAA C 
Rep-C_gRNA1-2: TCG TCT CAA GAA AAT GAA GAG TTT TAG AGC TAG AAA TAG C
Csy-D_gRNA1-3: TCG TCT CAA TCC TCC ACA ACC TGC CTA TAC GGC AGT GAA C
Rep-C_gRNA1-3: TCG TCT CAG GAT CTA CCA GAG TTT TAG AGC TAG AAA TAG C

**3plex-II**

Csy4-B_gRNA2-1: TCG TCT CCT GCA TGT GCT GCC TGC CTA TAC GGC AGT GAA CCT G
Rep-C_gRNA2-1: TCG TCT CAT GCA ATA GCA ACG TTT TAG AGC TAG AAA TAG C
Csy-D_gRNA2-2: TCG TCT CAT AAG TTC GAT TCC TGC CTA TAC GGC AGT GAA C
Rep-C_gRNA2-2: TCG TCT CAC TTA AGG CTC AAG TTT TAG AGC TAG AAA TAG C
Csy-D_gRNA2-3: TCG TCT CAA GAG AAG TCT TCC TGC CTA TAC GGC AGT GAA C
Rep-C_gRNA2-3: TCG TCT CAC TCT TCC CGA GTG TTT TAG AGC TAG AAA TAG C

**3plex-III**
Csy4-B_gRNA3-1: TCG TCT CCC CGA AGG CCA TCC TGC CTA TAC GGC AGT GAA CCT G

Rep-C_gRNA3-1: TCG TCT CAT CGG CGA TAA AAG TTT TAG AGC TAG AAA TAG C
Csy-D_gRNA3-2: TCG TCT CAT AAG CCA CAA ACC TGC CTA TAC GGC AGT GAA C
Rep-C_gRNA3-2: TCG TCT CAC TTA GTG TCA ATG TTT TAG AGC TAG AAA TAG C
Csy-D_gRNA3-3: TCG TCT CAA TTG ATA GTG CCC TGC CTA TAC GGC AGT GAA C
Rep-C_gRNA3-3: TCG TCT CAC AAT CGT TCG AAG TTT TAG AGC TAG AAA TAG C

**Universal primers from the promoter and terminater for all three constructs**

CmYLCV_pro: TGC TCT TCG CGC TGG CAG ACA TAC TGT CCC AC
Csy-E: TGC TCT TCT GAC CTG CCT ATA CGG CAG TGA AC

**Table S2.** Assembly of gRNA spacers by PCR amplification

3plex-I:
Reaction #1: CmY LCV _pro + Csy4_B gRNA1-1

Reaction #2: Rep_C gRNA1-1 + Csy_D gRNA1-2

Reaction #3: Rep_C gRNA1-2 + Csy_D gRNA1-3

Reaction #4: Rep_C gRNA1-3 + Csy_E

3plex-II:

Reaction #1: CmYLCV_pro + Csy4_B gRNA2-1

Reaction #2: Rep_C gRNA2-1 + Csy_D gRNA2-2

Reaction #3: Rep_C gRNA2-2 + Csy_D gRNA2-3

Reaction #4: Rep_C gRNA2-3 + Csy_E

3plex-III:

Reaction #1: CmYLCV_pro + Csy4_B gRNA3-1

Reaction #2: Rep_C gRNA3-1 + Csy_D gRNA3-2

Reaction #3: Rep_C gRNA3-2 + Csy_D gRNA3-3

Reaction #4: Rep_C gRNA3-3 + Csy_E

**Table S3.** Oligos for amplifying transgenes to detect transgenic plants; oligos for amplifying target genes and/or for CAPS PCR

**Oligos for amplifying transgenes sequences:**

GmUbi-ATG125: GATTCTATTGCCGTGGATTAGGG

AtCas9rev1: CTTCTTAGATGGAACCTTGTAC

BAR_F1: GTCTGCACCATCGTCAACCACTAC

BAR_R1: GGCGTTGCGTGCCTTCCAGGGGCC

**Oligos for amplifying target genes:**

LE F1: GTACCCAATAATGCTAGTATAA

LE R1: AACAATGACAATCAGTAGCGATC

KTi3 F1: ATGAAGGTAAGGCTCTTGAAA

KTi3 R1: TTTAGTGTAATGATTTCGTACAAC

P34 F1: CAGAAACAGGTGTCTTCACTGTT; (specific to 3plex-I)

P34 R1: CACTAATAAGGTTAGTTTGGTAA; (specific to 3plex-I)

P34 F2/3: TTACCAAACTAACCTTATTAGTG (specific to 3plex-II and III)

P34 R2/3: GTTTCATATCCGTCAATTGTAAC (specific to 3plex-II and III)

**Tables S4-S5 can be found in the supplemental .csv files accompanying this paper.**

**Table S6.** Least squared means for yield and maturity date for five triple-knockout lines and three checks grown in four Minnesota environments.

| Genotype | Yield (kg ha-1) | Maturity date (Days after Aug 31) |
| --- | --- | --- |
| Bert | 533.3 | 18.8 |
| WPT 673-7-8-8 | 501.8 | 20.5 |
| WPT 673-7-12-5 | 534.7 | 20.3 |
| MN0811CN | 617.7 | 7.6 |
| MN0811CN-BC4TN | 588.3 | 6.2 |
| M07-292111-BC4TN2 | 670.9 | 20.3 |
| M07-292111-BC4TN1 | 648.6 | 10.1 |
| MN1410 | 653.9 | 14.8 |
| LSD (0.05) | 35.8 | 1.3 |

**Table S7.** Comparison of least square (LS) means of yield (bushels/acre) for eight triple-knockout lines (above) grown in four Minnesota environments (below) using Tukey's Honestly Significant Differences (HSD).^1^

|  | Levels^2^ | LS Mean | Std Error |
| --- | --- | --- | --- |
| *Line* |  |  |  |
| 401-64-56TN | A | 60.9 | 3.9 |
| MN1410 | A | 59.4 | 3.1 |
| 401-90-114TN | A B | 58.9 | 3.1 |
| M08-362045L | A B | 56.1 | 3.1 |
| 404-49-292TN | B | 53.4 | 3.1 |
| 673-7-12-5 | A | 48.6 | 3.1 |
| Bert | A | 48.4 | 3.1 |
| 673-7-8-8 | A | 45.6 | 3.1 |
| *Environment* |  |  |  |
| SP_2020 | A | 79.8 | 2.1 |
| RO_2020 | B | 55.0 | 2.1 |
| BE_2021 | C | 43.0 | 2.1 |
| RO_2021 | D | 37.7 | 2.7 |

^1^ Backcross-derived lines '401-64-56-TN', '401-90-114TN', and '404-49-292TN' were compared to their backcross parents 'MN1410' and 'M08-362045L'. CRISPR-derived lines were compared to their non-transgenic parent line 'Bert'.

^2^ Lines or environments sharing the same letter are not significantly different.

**Table S8.** Analysis of Variance for the components of field-based yield trials for five triple-knockout mutant lines and three parental lines grown in four Minnesota environments (year by location).

| Source | DF | Sum of Squares | Mean Square | F Ratio | Prob > F |
| --- | --- | --- | --- | --- | --- |
| *Model Analysis of Variance* |  |  |  |  |  |
| Model | 31 | 30123.5 | 971.7 | 35.0 | <.0001 |
| Error | 86 | 2387.9 | 27.8 |  |  |
| Corrected Total | 117 | 32511.4 |  |  | Adjusted R-Square: 0.9 |
| *Test of Model Effects** |  |  |  |  |  |
| Entry (f) | 7 | 3212.9 | 459.0 | 3.2 | 0.0166 |
| Environment (r) | 3 | 24168.7 | 8056.2 | 56.4 | <.0001 |
| Environment x Entry (r) | 21 | 3059.02 | 145.7 | 5.2 | <.0001 |

*Indicates model term as either a fixed effect (f), or random effect (r)
