# Supplementary material for: Genomic and biochemical comparison of allelic triple‐mutant lines derived from conventional breeding and multiplex gene editing
Source: Plant Genome. 2025 Jun 5;18(2):e70056. doi: 10.1002/tpg2.70056 (PMC12141651; doi:10.1002/tpg2.70056)
Supplement: Supplementary file 2 — Table S1. Oligos for cloning 3plex gRNAs Table S2. Assembly of gRNA spacers by PCR amplification Table S3. Oligos for amplifying transgenes to detect transgenic plants; oligos for amplifying target genes and/or for CAPS PCR Table S6. Least squared means for yield and maturity date for five triple‐knockout lines and three checks grown in four Minnesota environments. Table S7. Comparison of least square (LS) means of yield (bushels/acre) for eight triple‐knockout lines (above) grown in four Minnesota environments (below) using Tukey's Honestly Significant Differences (HSD).1 Table S8. Analysis of Variance for the components of field‐based yield trials for five triple‐knockout mutant lines and three parental lines grown in four Minnesota environments (year by location). [file TPG2-18-e70056-s002.docx]

**Table S1.** Oligos for cloning 3plex gRNAs

**3plex-I**

Csy4-B_gRNA1-1: TCG TCT CCT TTT GAT GGA TCC TGC CTA TAC GGC AGT GAA CCT G
Rep-C_gRNA1-1: TCG TCT CAA AAA CGA CGT CTG TTT TAG AGC TAG AAA TAG C
Csy-D_gRNA1-2: TCG TCT CAT TCT TGT TGG CCC TGC CTA TAC GGC AGT GAA C 
Rep-C_gRNA1-2: TCG TCT CAA GAA AAT GAA GAG TTT TAG AGC TAG AAA TAG C
Csy-D_gRNA1-3: TCG TCT CAA TCC TCC ACA ACC TGC CTA TAC GGC AGT GAA C
Rep-C_gRNA1-3: TCG TCT CAG GAT CTA CCA GAG TTT TAG AGC TAG AAA TAG C

**3plex-II**

Csy4-B_gRNA2-1: TCG TCT CCT GCA TGT GCT GCC TGC CTA TAC GGC AGT GAA CCT G
Rep-C_gRNA2-1: TCG TCT CAT GCA ATA GCA ACG TTT TAG AGC TAG AAA TAG C
Csy-D_gRNA2-2: TCG TCT CAT AAG TTC GAT TCC TGC CTA TAC GGC AGT GAA C
Rep-C_gRNA2-2: TCG TCT CAC TTA AGG CTC AAG TTT TAG AGC TAG AAA TAG C
Csy-D_gRNA2-3: TCG TCT CAA GAG AAG TCT TCC TGC CTA TAC GGC AGT GAA C
Rep-C_gRNA2-3: TCG TCT CAC TCT TCC CGA GTG TTT TAG AGC TAG AAA TAG C

**3plex-III**
Csy4-B_gRNA3-1: TCG TCT CCC CGA AGG CCA TCC TGC CTA TAC GGC AGT GAA CCT G

Rep-C_gRNA3-1: TCG TCT CAT CGG CGA TAA AAG TTT TAG AGC TAG AAA TAG C
Csy-D_gRNA3-2: TCG TCT CAT AAG CCA CAA ACC TGC CTA TAC GGC AGT GAA C
Rep-C_gRNA3-2: TCG TCT CAC TTA GTG TCA ATG TTT TAG AGC TAG AAA TAG C
Csy-D_gRNA3-3: TCG TCT CAA TTG ATA GTG CCC TGC CTA TAC GGC AGT GAA C
Rep-C_gRNA3-3: TCG TCT CAC AAT CGT TCG AAG TTT TAG AGC TAG AAA TAG C

**Universal primers from the promoter and terminater for all three constructs**

CmYLCV_pro: TGC TCT TCG CGC TGG CAG ACA TAC TGT CCC AC
Csy-E: TGC TCT TCT GAC CTG CCT ATA CGG CAG TGA AC

**Table S2.** Assembly of gRNA spacers by PCR amplification

3plex-I:
Reaction #1: CmY LCV _pro + Csy4_B gRNA1-1

Reaction #2: Rep_C gRNA1-1 + Csy_D gRNA1-2

Reaction #3: Rep_C gRNA1-2 + Csy_D gRNA1-3

Reaction #4: Rep_C gRNA1-3 + Csy_E

3plex-II:

Reaction #1: CmYLCV_pro + Csy4_B gRNA2-1

Reaction #2: Rep_C gRNA2-1 + Csy_D gRNA2-2

Reaction #3: Rep_C gRNA2-2 + Csy_D gRNA2-3

Reaction #4: Rep_C gRNA2-3 + Csy_E

3plex-III:

Reaction #1: CmYLCV_pro + Csy4_B gRNA3-1

Reaction #2: Rep_C gRNA3-1 + Csy_D gRNA3-2

Reaction #3: Rep_C gRNA3-2 + Csy_D gRNA3-3

Reaction #4: Rep_C gRNA3-3 + Csy_E

**Table S3.** Oligos for amplifying transgenes to detect transgenic plants; oligos for amplifying target genes and/or for CAPS PCR

**Oligos for amplifying transgenes sequences:**

GmUbi-ATG125: GATTCTATTGCCGTGGATTAGGG

AtCas9rev1: CTTCTTAGATGGAACCTTGTAC

BAR_F1: GTCTGCACCATCGTCAACCACTAC

BAR_R1: GGCGTTGCGTGCCTTCCAGGGGCC

**Oligos for amplifying target genes:**

LE F1: GTACCCAATAATGCTAGTATAA

LE R1: AACAATGACAATCAGTAGCGATC

KTi3 F1: ATGAAGGTAAGGCTCTTGAAA

KTi3 R1: TTTAGTGTAATGATTTCGTACAAC

P34 F1: CAGAAACAGGTGTCTTCACTGTT; (specific to 3plex-I)

P34 R1: CACTAATAAGGTTAGTTTGGTAA; (specific to 3plex-I)

P34 F2/3: TTACCAAACTAACCTTATTAGTG (specific to 3plex-II and III)

P34 R2/3: GTTTCATATCCGTCAATTGTAAC (specific to 3plex-II and III)

**Tables S4-S5 can be found in the supplemental .csv files accompanying this paper.**

**Table S6.** Least squared means for yield and maturity date for five triple-knockout lines and three checks grown in four Minnesota environments.

| Genotype | Yield (kg ha-1) | Maturity date (Days after Aug 31) |
| --- | --- | --- |
| Bert | 533.3 | 18.8 |
| WPT 673-7-8-8 | 501.8 | 20.5 |
| WPT 673-7-12-5 | 534.7 | 20.3 |
| MN0811CN | 617.7 | 7.6 |
| MN0811CN-BC4TN | 588.3 | 6.2 |
| M07-292111-BC4TN2 | 670.9 | 20.3 |
| M07-292111-BC4TN1 | 648.6 | 10.1 |
| MN1410 | 653.9 | 14.8 |
| LSD (0.05) | 35.8 | 1.3 |

**Table S7.** Comparison of least square (LS) means of yield (bushels/acre) for eight triple-knockout lines (above) grown in four Minnesota environments (below) using Tukey's Honestly Significant Differences (HSD).^1^

|  | Levels^2^ | LS Mean | Std Error |
| --- | --- | --- | --- |
| *Line* |  |  |  |
| 401-64-56TN | A | 60.9 | 3.9 |
| MN1410 | A | 59.4 | 3.1 |
| 401-90-114TN | A B | 58.9 | 3.1 |
| M08-362045L | A B | 56.1 | 3.1 |
| 404-49-292TN | B | 53.4 | 3.1 |
| 673-7-12-5 | A | 48.6 | 3.1 |
| Bert | A | 48.4 | 3.1 |
| 673-7-8-8 | A | 45.6 | 3.1 |
| *Environment* |  |  |  |
| SP_2020 | A | 79.8 | 2.1 |
| RO_2020 | B | 55.0 | 2.1 |
| BE_2021 | C | 43.0 | 2.1 |
| RO_2021 | D | 37.7 | 2.7 |

^1^ Backcross-derived lines '401-64-56-TN', '401-90-114TN', and '404-49-292TN' were compared to their backcross parents 'MN1410' and 'M08-362045L'. CRISPR-derived lines were compared to their non-transgenic parent line 'Bert'.

^2^ Lines or environments sharing the same letter are not significantly different.

**Table S8.** Analysis of Variance for the components of field-based yield trials for five triple-knockout mutant lines and three parental lines grown in four Minnesota environments (year by location).

| Source | DF | Sum of Squares | Mean Square | F Ratio | Prob > F |
| --- | --- | --- | --- | --- | --- |
| *Model Analysis of Variance* |  |  |  |  |  |
| Model | 31 | 30123.5 | 971.7 | 35.0 | <.0001 |
| Error | 86 | 2387.9 | 27.8 |  |  |
| Corrected Total | 117 | 32511.4 |  |  | Adjusted R-Square: 0.9 |
| *Test of Model Effects** |  |  |  |  |  |
| Entry (f) | 7 | 3212.9 | 459.0 | 3.2 | 0.0166 |
| Environment (r) | 3 | 24168.7 | 8056.2 | 56.4 | <.0001 |
| Environment x Entry (r) | 21 | 3059.02 | 145.7 | 5.2 | <.0001 |

*Indicates model term as either a fixed effect (f), or random effect (r)
